# Aetiology-Specific Estimates of the Global and Regional Incidence and Mortality of Diarrhoeal Diseases Commonly Transmitted through Food

**DOI:** 10.1371/journal.pone.0142927

**Published:** 2015-12-03

**Authors:** Sara M. Pires, Christa L. Fischer-Walker, Claudio F. Lanata, Brecht Devleesschauwer, Aron J. Hall, Martyn D. Kirk, Ana S. R. Duarte, Robert E. Black, Frederick J. Angulo

**Affiliations:** 1 National Food Institute, Danish Technical University, Copenhagen, Denmark; 2 Johns Hopkins University, Baltimore, United States of America; 3 Instituto de Investigación Nutricional, Lima, Peru; 4 US Naval Medical Research Unit No. 6, Callao, Peru; 5 Faculty of Veterinary Medicine, Ghent University, Merelbeke, Belgium; Institute of Health and Society (IRSS), Faculty of Public Health, Université Catholique de Louvain, Brussels, Belgium; Institute of Tropical Medicine, Antwerp, Belgium; 6 Centers for Disease Control and Prevention, Atlanta, Georgia, United States of America; 7 The Australian National University, Canberra, Australia; Curtin University, AUSTRALIA

## Abstract

**Background:**

Diarrhoeal diseases are major contributors to the global burden of disease, particularly in children. However, comprehensive estimates of the incidence and mortality due to specific aetiologies of diarrhoeal diseases are not available. The objective of this study is to provide estimates of the global and regional incidence and mortality of diarrhoeal diseases caused by nine pathogens that are commonly transmitted through foods.

**Methods and Findings:**

We abstracted data from systematic reviews and, depending on the overall mortality rates of the country, applied either a national incidence estimate approach or a modified Child Health Epidemiology Reference Group (CHERG) approach to estimate the aetiology-specific incidence and mortality of diarrhoeal diseases, by age and region. The nine diarrhoeal diseases assessed caused an estimated 1.8 billion (95% uncertainty interval [UI] 1.1–3.3 billion) cases and 599,000 (95% UI 472,000–802,000) deaths worldwide in 2010. The largest number of cases were caused by norovirus (677 million; 95% UI 468–1,153 million), enterotoxigenic *Escherichia coli* (ETEC) (233 million; 95% UI 154–380 million), *Shigella* spp. (188 million; 95% UI 94–379 million) and *Giardia lamblia* (179 million; 95% UI 125–263); the largest number of deaths were caused by norovirus (213,515; 95% UI 171,783–266,561), enteropathogenic *E*. *coli* (121,455; 95% UI 103,657–143,348), ETEC (73,041; 95% UI 55,474–96,984) and *Shigella* (64,993; 95% UI 48,966–92,357). There were marked regional differences in incidence and mortality for these nine diseases. Nearly 40% of cases and 43% of deaths caused by these nine diarrhoeal diseases occurred in children under five years of age.

**Conclusions:**

Diarrhoeal diseases caused by these nine pathogens are responsible for a large disease burden, particularly in children. These aetiology-specific burden estimates can inform efforts to reduce diarrhoeal diseases caused by these nine pathogens commonly transmitted through foods.

## Introduction

Diarrhoeal diseases are a major cause of disease burden worldwide [1]. The Global Burden of Disease Study 2010 (GBD 2010) ranked diarrhoeal diseases as the fourth largest disease burden, accounting for 3.6% of the total disease burden globally. Diarrhoeal diseases accounted for an even higher proportion (5%) of the total disease burden in children <5 years of age [1]. Diarrhoeal diseases have a substantially higher impact in low-income countries and regions with poor water quality, sanitation and food safety.

Diarrhoeal diseases are caused by a variety of bacteria, viruses, and parasites, many of which are commonly transmitted through food [2]. Despite the large disease burden caused by these pathogens, the global contribution of specific aetiological agents of diarrhoeal diseases is largely unknown. For example, recent studies have estimated the worldwide incidence of diarrhoeal diseases in children <5 years of age [3], and in older children and adults [4], but did not provide aetiology-specific estimates. Lanata *et al*. [5] and Fisher Walker *et al*. [6] provided aetiology-specific estimates, but only for diarrhoeal deaths among children <5 years of age, and diarrhoeal cases in persons ≥5 years of age, respectively. Another large-scale study estimated diarrhoeal aetiologies in children <5 years of age in specific study sites, particularly in sub-Saharan Africa and South Asia [7]. Other studies have estimated the incidence of specific foodborne diseases, many of which cause diarrhoea, but each focused on a single developed country [8–14].

To identify and prioritize targeted interventions to reduce the public health impact of foodborne diseases, public health policy makers and other stakeholders need aetiology-specific regional and global estimates of the incidence and mortality of diarrhoeal diseases caused by pathogens that are commonly transmitted through foods. These estimates, combined with knowledge on the proportion of this burden that is derived from foods, will form the basis for the estimation of the global and regional burden of foodborne diseases.

As part of the effort by the World Health Organization (WHO) Foodborne Disease Burden Epidemiology Reference Group (FERG; http://www.who.int/foodsafety/areas_work/foodborne-diseases/ferg/en/) to estimate the disease burden of foodborne diseases, we estimated the global and regional incidence and mortality of diarrhoeal diseases which are commonly transmitted through foods.

## Methods

After reviewing the epidemiology of diseases caused by bacteria, viruses, and protozoa that are commonly transmitted to humans through food, and which are included in FERG, we selected nine pathogens which commonly cause diarrhoea for inclusion in our study: *Campylobacter* spp., *Cryptospordium* spp., *Entamoeba histolytica*, enterotoxigenic *Escherichia coli* (ETEC), enteropathogenic *E*. *coli* (EPEC), *Giardia lamblia*, norovirus, *Salmonella* spp., and *Shigella* spp. We did not include Shiga-toxin producing *E*. *coli* (STEC), and *Vibrio cholerae*, diarrhoeal pathogens included in FERG, because the incidence and mortality of diseases caused by these agents have been estimated using different approaches and published elsewhere [15, 16]. Although some countries have published national incidence and mortality estimates for diseases caused by most of these nine pathogens, such estimates of foodborne diseases, which are considered to be the highest quality burden estimates for these diseases [17], are only available from a limited number of countries. We therefore used two approaches to estimate the incidence and mortality of the diseases caused by the nine selected diarrhoeal pathogens, and applied one or the other approach in each country in the six regions. Approach 1 was applied to all countries in the European Region (EURO) and all other countries with overall low mortality as defined by WHO (http://www.who.int/choice/demography/mortality_strata/en/); approach 2 was applied to all remaining countries. Using these two approaches allowed us to utilize the highest quality available data in countries with overall low mortality which have similar sanitary and public health infrastructure and presumed incidence of foodborne diseases. The final results of the two approaches were merged in a last step to determine global estimates and estimates for the 6 regions.

### Approach 1 –EURO countries and other low-mortality countries

The first approach utilized available national incidence and mortality estimates of diseases caused by the nine selected pathogens and was applied to 61 countries in EURO (sub-regions A, B, and C), and low-mortality countries in American Region (AMRO; sub-region A) and Western Pacific Region (WPRO; sub-region A). We conducted a literature review and consulted with the International Collaboration of Foodborne Disease Burden of Illness Studies (http://www.cdc.gov/ncezid/dfwed/international/enteric-burden-collaboration.html) to identify all published national estimates (with associated uncertainty intervals) of foodborne diseases. Such estimates were derived through studies that corrected national surveillance data to account for under-diagnosis and underreporting [8]. In cases where national estimates were expressed as number of cases and deaths, and not as incidence and mortality rates (per 100,000 population), we used United Nations population figures for the national study year to create such rates. If a national estimate only included domestically-acquired infections, we used the proportion of infections acquired during international travel in a neighbouring country to derive revised estimates that included infections acquired abroad. We applied the national incidence and mortality estimates, with the associated uncertainty intervals, to each country with such national estimates, and the median incidence and mortality of all studies, with uncertainty intervals associated with those median estimates, to each country in EURO, AMRO sub-region A, and WPRO sub-region A that did not have national incidence or mortality estimates. This approach resulted in incidence and mortality estimates (per 100,000 population), with uncertainty intervals, for each of the 61 countries for each of the nine diseases. Overall estimates were partitioned to the age groups <5 years of age and ≥5 years of age on the basis of the age distribution of the population in each region. Further details of this approach are available in S1 Appendix.

### Approach 2 –All other countries

The second approach, based on a modification of the Child Health Epidemiology Reference Group (CHERG) method, was applied to the 133 remaining countries [5].

#### Regional incidence and mortality of diarrhoea

First, we identified the total number of diarrhoeal cases in the 133 countries for 2010 by combining estimates based on systematic reviews for children <5 years of age and persons ≥5 years of age [3,4]. For the estimate of the total number of diarrhoeal deaths, we obtained data on the total number of deaths in the 133 countries in 2010 attributed to diarrhoeal diseases from WHO (http://www.who.int/gho/en/; Accessed 6 June 2014); we used the range of diarrhoeal deaths estimated in the Global Burden of Disease 2010 study (GBD2010) to derive an uncertainty interval for the death envelope. We derived the final number of diarrhoeal cases (“diarrhoeal envelope”) and diarrhoeal deaths (“diarrhoeal death envelope”) by subtracting published estimates of the number of diarrhoeal cases and deaths caused by STEC [15] and *V*. *cholerae* [16].

#### Aetiology proportions

Our next step was to estimate the proportion of diarrhoeal illnesses and deaths due to the nine pathogens by extracting the aetiological proportions of diarrhoeal cases and deaths for each pathogen by region from systematic reviews of studies reporting stool sample isolation and detection from inpatient, outpatient, and community-based studies of persons with diarrhoea. We assumed that the distribution of pathogens observed among outpatient and community studies represented the pathogen prevalence among diarrhoeal cases, and that the distribution of pathogens among inpatients hospitalized for severe diarrhoea represented the pathogen prevalence among diarrhoeal deaths for all age groups. Due to data scarcity, we also included inpatient studies to estimate the aetiological proportions of diarrhoeal cases in persons ≥5 years of age (i.e. all available studies). We assumed that *G*. *lamblia* infection was unlikely to result in death based upon the data available from the national foodborne disease mortality estimates, and therefore excluded it from mortality estimates.

To determine aetiological proportions, we used 3 systematic reviews to identify studies that reported isolation or detection of pathogens from stool specimens or rectal swabs collected from persons with diarrhoea. For norovirus, we used a previously published systematic review including studies published between 1990 and 2012 and then updated through 2014 [17]. The norovirus-specific review was conducted because of the increased recent use of molecular diagnostics globally and as part of a parallel effort to estimate the global burden of norovirus disease [17]. For all other pathogens we included two previously published systematic reviews: 1) the aetiology of diarrhoeal disease studies for children ≥5 years of age published between 1980 and 2008 [3] and 2) the aetiology among children <5 years of age published between 1990 and 2011 [5]. We updated each of these reviews to include studies published through 2012. S2 Appendix details the systematic review methodologies and results of the updated reviews, including search terms and inclusion/exclusion criteria. Studies focusing only on norovirus collected in the general reviews were excluded to avoid duplicates with the norovirus-specific review. The general systematic reviews collected data on isolation or detection of the nine pathogens included in our study and on pathogens not commonly transmitted by food (e.g. rotavirus, sapovirus, astrovirus, and coronavirus); the latter were grouped together as “other pathogens”.

We initially calculated study-specific aetiology-proportions by dividing the number of samples positive for the pathogen by the total number of samples tested in that study (*study-proportions*); we then estimated regional proportions by calculating the median aetiology-proportion of all study-estimates within each region (*regional-proportions*). For example, if 10 studies conducted in the Western Pacific region (WPRO) provided data on the number of *Salmonella* isolates among diarrhoeal cases, this region’s *Salmonella*-proportion was estimated as the median of the estimated 10 study-specific aetiology proportions. Since several of the studies among children <5 years of age used narrower age ranges, in analyses *a* and *b* we calculated an age-adjusted proportion for this age group by calculating a conversion factor as the ratio of the median proportion in the age group 0–59 months to the median proportion in age group X ((median (prev0–59)/median (prevX)). We applied this approach when three or more studies for each pathogen contributed to each of the two medians. If this condition was not met, we borrowed the conversion factor for the age group from a similar age group within the same pathogen (for example, used the conversion factor calculated for studies including infants 0–11 months of age for studies that included infants 0–5 months of age). For persons ≥5 years of age, we have assumed that differences between narrower age categories would be diluted in this very broad population group and have chosen not to age-adjust medians. To estimate the proportion of diarrhoeal stools due to unknown aetiology, we included studies that sought ≥8 pathogens and reported patients with an unknown cause of disease.

If only one study tested for a given pathogen in one region, we applied criteria to identify outliers and prevent potentially non-representative studies from misrepresenting the final regional aetiology-proportions. An outlier was defined as a regional estimate ±5 times the global median. If a regional estimate derived from ≥2 studies was identified as an outlier, the individual studies’ prevalence and sample size were evaluated, and a particular study was excluded if it represented an outlier when compared to remaining studies within the region. Outliers were excluded from the results and taken as a missing value. If a regional median aetiological proportion estimate was missing (due to either missing data or exclusion of an outlier), the missing regional estimate was replaced by the global median of the aetiological proportions for that pathogen. All global medians were estimated after the exclusion of potential outliers.

#### Statistical analysis

To account for uncertainty in these calculations, we used Monte Carlo simulation in all steps of the analysis. We applied a bootstrapping analysis to derive 95% uncertainty intervals (UI) around aetiology-proportions. ‘Pseudo-data sets’ were created by sampling each study with replacement from the real dataset. In each of these 1,000 pseudo-datasets, a different random number of positive samples for each study was generated from a binomial distribution defined on the basis of the number of samples and the expected proportion in that study. All pseudo-datasets were used in the estimation procedure described above to generate corresponding 1,000 study-specific aetiology proportions, and regional aetiology proportions. The 2.5th and 97.5th percentile of these proportions gave the 95% UI. Data management and analyses were conducted in SAS Enterprise Guide 4.3.

We then constrained the aetiological proportions for the nine diarrhoeal pathogens, for other pathogens not commonly transmitted by food, and unknown aetiology to ensure that they did not sum to more than 100% in each region. For this purpose, we first fitted univariate Beta(α, β) distributions to the median proportions and simulated quantiles (i.e., the 2.5th and 97.5th percentiles). The distributions’ optimized parameters were estimated via one dimensional optimization, minimizing the squared distance between the estimated and fitted quantiles, and ensuring the median of the fitted distribution to be similar to the estimated median. Next, 10,000 random deviates were sampled from the fitted Beta distributions. If needed, an "unknown aetiology" category was created as 1 minus the sum of simulated proportions across aetiologies, per iteration. Finally, the random deviates were normalized iteration-wise by dividing them by the sum of simulated aetiological fractions.

Once estimates of aetiological proportions for cases and deaths for the nine pathogens were derived, the regional aetiological proportions for each disease were multiplied by the respective estimates for total diarrhoeal illnesses and deaths in those regions, accounting for uncertainty using a stochastic model with 10,000 iterations. We then derived age-stratified incidence and mortality estimates, with associated uncertainty, for each disease for each region using country-level population data for 2010 from the 2012 Revision of the United Nations World Population Prospects (esa.un.org/wpp, accessed June 10, 2014); among the 133 countries, all countries in the same region were assumed to have the same incidence and mortality, and associated uncertainty, for each pathogen. In order to aggregate results with estimates derived for low-mortality countries (*approach 1*), age-specific estimates were then grouped into overall estimates. The stochastic model was implemented in R version 3.1.1 (R Core Team, 2014).

#### Global estimates (combining Approach 1 and Approach 2)

The incidence and mortality estimates, with associated uncertainty, for each disease for each region obtained through approach 1 and approach 2 were combined in a final step to produce final global estimates and estimates for each of the six regions.

## Results

### Approach 1 –EURO countries and other low-mortality countries

We identified national incidence and mortality estimates of the nine diarrhoeal diseases for seven countries: Australia, Canada, France, Netherlands, New Zealand, United States of America, and the United Kingdom [8–14]. The national estimates from Australia and Canada included only domestically-acquired infections; revised national estimates were derived using data on international travel-acquired infections from the New Zealand and the United States, respectively. Estimates of incidence and mortality for each of 7 countries are available in S1 Appendix.

### Approach 2 –All other countries

**Fig 1:**
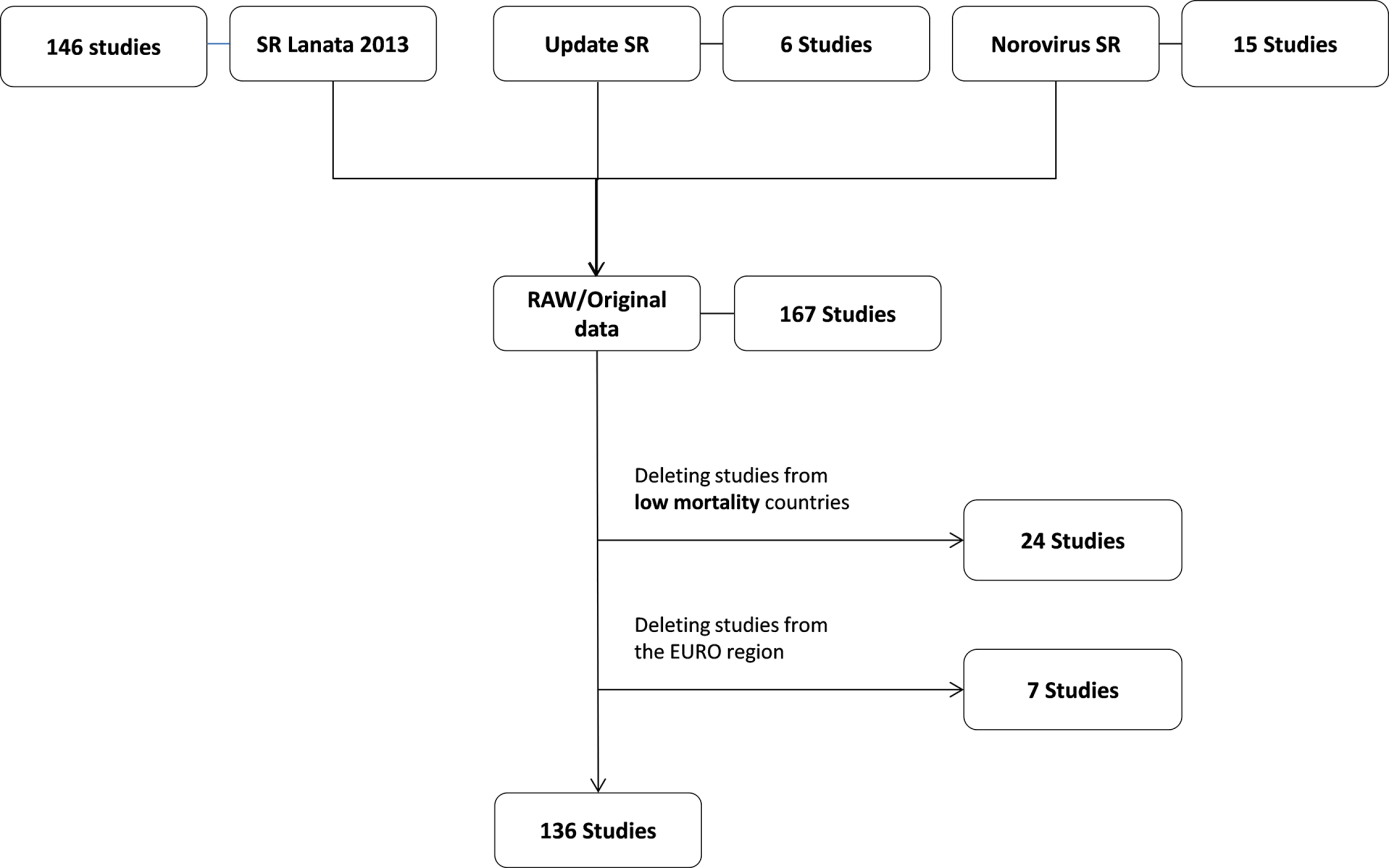
Overview of data utilized to estimate the aetiology-proportions of diarrhoeal cases in children <5 years of age (outpatients and community studies). Studies conducted in EURO Region countries and other low-mortality countries (sub-region A) were removed from the original data set. *SR: systematic review. SR Lanata 2013 [5]; Update SR: see S2 Appendix; Norovirus SR [14].

**Fig 2:**
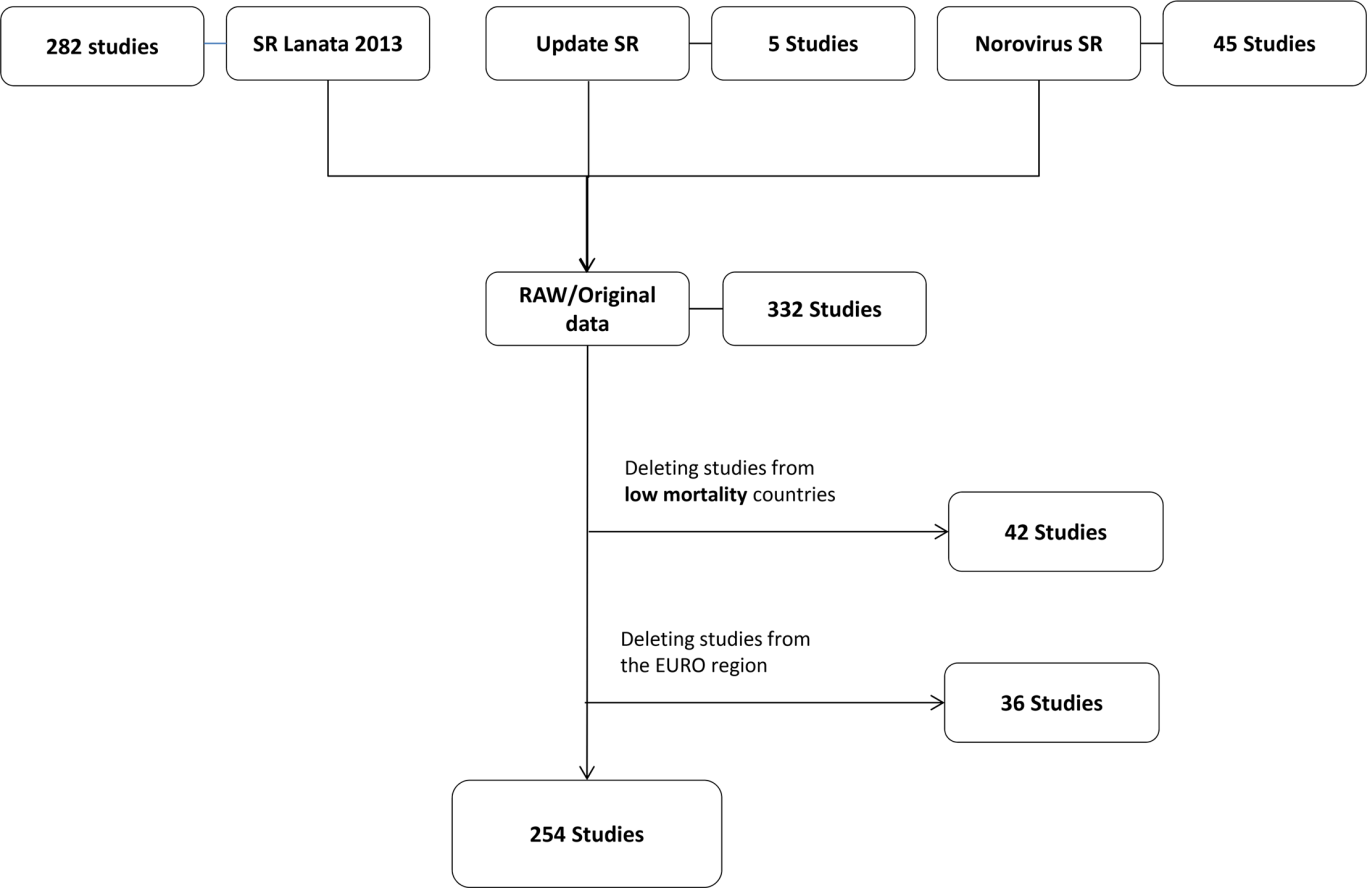
Overview of data utilized to estimate the aetiology-proportions of diarrhoeal deaths in children <5 years of age (inpatient studies). Studies conducted in EURO Region countries and other low-mortality countries (sub-region A) were removed from the original data set. *SR: systematic review. SR Lanata 2013 [5]; Update SR: see S2 Appendix; Norovirus SR [14].

**Fig 3:**
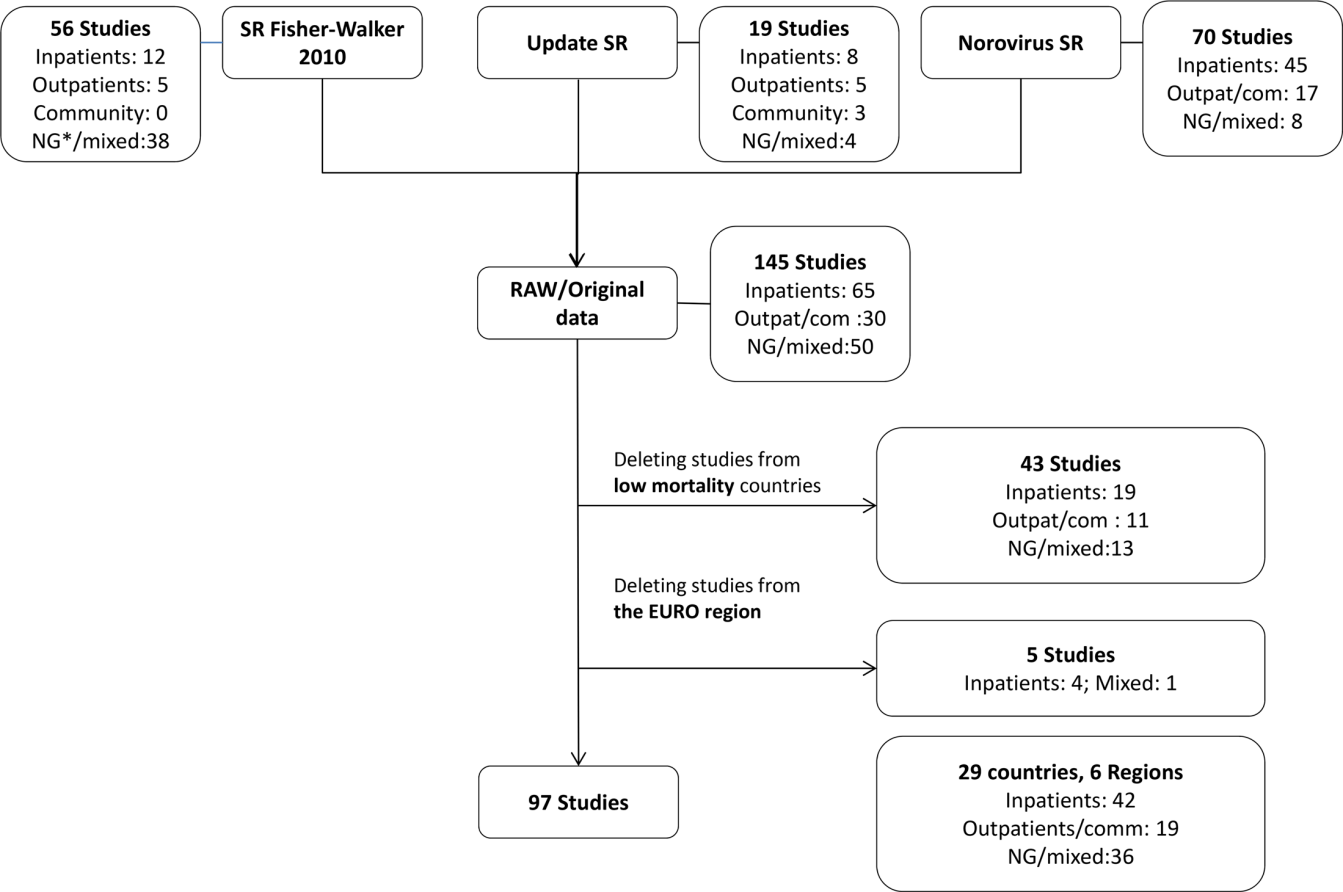
Overview of data utilized to estimate the aetiology-proportions of diarrhoeal cases and deaths in persons >5 years of age (inpatients, outpatients and community studies). Studies conducted in EURO Region countries and other low-mortality countries (sub-region A) were removed from the original data set. *NG: information on type of patients included in the study not given; SR: systematic review; SR Fisher-Walker 2010 [6]; Update SR: see S2 Appendix; Norovirus SR [14].

The systematic reviews conducted to collect data to estimate the proportion of diarrhoeal illnesses and deaths in the 133 other countries yielded 136 outpatient and community studies (Fig 1) and 254 inpatient studies (Fig 2) among children <5 years of age, and 97 inpatient, outpatient, and community studies (Fig 3) among persons ≥5 years of age. Table 1 lists the number of studies for each of the nine pathogens by region. The final number of studies providing data for individual pathogens in different regions varied substantially. The data available for persons ≥5 years of age were scarcer and the number of studies for each region was low, particularly for the AFRO and AMRO regions. Table A in S1 File presents the estimates of the regional envelopes of diarrhoeal cases and deaths, and Tables B1 to B4 in S1 File present the contribution of each pathogen for these envelopes.

**Table 1:** Number of studies IDENTIFIED for each pathogen in each region^†^.

A. Studies among children <5 years of age												
	Community and outpatient studies (136 total*)—for calculating cases						Inpatient studies (254 total*)—for calculating deaths					
	AFRO	AMRO	EMRO	SEARO	WPRO	TOTAL	AFRO	AMRO	EMRO	SEARO	WPRO	TOTAL
*Salmonella*	8	11	3	7	3	32	2	11	2	9	1	25
*Campylobacter*	10	13	7	7	1	38	2	9	1	10	1	23
ETEC	6	14	6	5	1	32	1	9	3	8	0	21
EPEC	3	7	0	3	1	14	1	5	2	2	0	10
*Shigella*	8	17	5	10	3	43	2	14	2	11	1	30
*Cryptosporidium*	4	9	1	3	1	18	2	12	0	10	1	25
*Giardia*	7	11	2	6	2	28	1	10	0	4	1	16
*Entamoeba histolytica*	7	6	2	6	2	23	1	7	0	5	1	14
Norovirus	0	3	1	3	1	8	3	4	2	9	13	31
B. Studies among persons ≥5 years of age												
	Community, outpatient, inpatient studies (97 total*)—for calculating cases						Inpatient studies (42 total*)—for calculating deaths					
	AFRO	AMRO	EMRO	SEARO	WPRO	TOTAL	AFRO	AMRO	EMRO	SEARO	WPRO	TOTAL
*Salmonella*	3	1	3	4	5	16	0	1	1	1	2	5
*Campylobacter*	2	1	6	3	5	17	0	1	1	0	2	4
ETEC	2	1	1	4	4	12	0	0	0	1	1	2
EPEC	1	1	2	2	2	8	0	1	0	0	0	1
*Shigella*	3	0	6	10	9	28	0	0	1	1	2	4
*Cryptosporidium*	1	1	1	6	1	10	0	0	0	1	2	3
*Giardia*	2	0	1	5	3	11	0	0	0	0	1	1
*Entamoeba histolytica*	2	0	0	6	3	11	0	0	0	0	1	1
Norovirus	3	4	7	4	22	40	3	3	4	1	13	24

*Not all studies provide data for pathogens included in our analysis. Remaining studies were used to estimate the aetiology-proportion of "others".

†Studies conducted in countries in the EURO region, AMRO sub-region A, or WPRO sub-region A were not included in the analyses.

### Global estimates

We estimated that the nine pathogens included in our study resulted in a total of 4.6 billion cases (95% UI 3.5–6.5) and 1.6 million deaths (95% UI 1.3–1.9) in 2010 worldwide (Table 2). In the 61 countries in EURO and AMRO and WPRO A regions, we estimated that these nine pathogens resulted in 852.2 million cases (95% UI 649.8–1,095.7) and 23,898 deaths (95% UI 14,607–34,212. In the remaining 133 countries, we estimated these pathogens resulted in a total of 3.4 billion cases (95% UI 1.4–8.4 billion) and 1,560,986 deaths in 2010 (95% UI 1,322,987–1,845,688). Global and regional estimates of incidence and mortality rates caused by these pathogens are presented in S1 Table. Globally, among the diseases caused by the nine pathogens, the largest number of cases were caused by norovirus (677 million; 95% UI 468–1,153 million), ETEC (233 million; 95% UI 154–380 million), *Shigella* spp. (188 million; 95% UI 94–379 million) and *G*. *lamblia* (179 million; 95% UI 125–263); the largest number of deaths were caused by norovirus (213,515; 95% UI 171,783–266,561), EPEC (121,455; 95% UI 103,657–143,348), ETEC (73,041; 95% UI 55,474–96,984) and *Shigella* spp. (64,993; 95% UI 48,966–92,357). Nearly 40% of cases and 43% of disease deaths caused by these nine pathogens occurred in children <5 years of age.

**Table 2:** Total global cases and deaths of 10 foodborne diarrhoeal pathogens (median and 95% Uncertainty Intervals (UI)).

Pathogen	ILLNESSES			DEATHS		
	61 countries in AMRO sub-region A, EURO and WPRO sub-region A)^	Remaining 133 countries^^	Total	61 countries in AMRO sub region A, EURO and WPRO sub region A)^	Remaining 133 countries^^	Total
*Campylobacter* *	10,260,163 (8,322,243–12,377,518)	155,814,695 (81,886,046–290,708,149)	166,175,078 (92,227,873–300,877,905)	760 (289–1,579)	35,483 (25,617–52,949)	36,300 (26,481–53,796)
EPEC	210,982 (95,774–363,569)	80,844,438 (40,503,794–171,242,419)	81,082,327 (40,716,656–171,419,480)	0 (0–0)‡	122,760 (97,115–154,869)	122,760 (97,115–154,869)
ETEC	210,362 (94,709–362,983)	240,672,835 (160,765,104–377,223,731)	240,886,759 (160,890,532–377,471,599)	0 (0–0)‡	73,857 (53,851–103,026)	73,857 (53,851–103,026)
Norovirus**	95,600,451 (73,123,103–119,377,846)	588,222,182 (396,697,200–1,024,027,674)	684,850,131 (490,930,402–1,122,947,359)	2,621 (2,047–3,255)	209,851 (157,982–275,639)	212,489 (160,595–278,420)
*Shigella*	849,468 (468,137–1,353,176)	190,006,150 (96,946,834–363,310,470)	190,849,501 (97,832,995–363,915,689)	296 (56–780)	65,472 (46,014–96,732)	65,796 (46,317–97,036)
*Salmonella* ***	4,575,153 (3,443,628–5,943,290)	148,479,623 (60,553,452–377,398,573)	153,097,991 (64,733,607–382,208,079)	2,680 (1,655–4,289)	54,227 (40,611–85,357)	56,969 (43,272–88,129)
*Cryptosporidium*	2,901,364 (1,534,357–5,101,235)	60,925,188 (40,122,467–101,826,587)	64,003,709 (43,049,455–104,679,951)	310 (78–742)	27,207 (18,237–44,265)	27,553 (18,532–44,654)
*Giardia*	5,438,836 (4,605,937–6,346,522)	178,421,363 (124,530,817–257,466,898)	183,842,615 (130,018,020–262,838,002)	NA^μ^	NA^μ^	NA^μ^
*Entamoeba histolytica*	NA†	103,943,952 (47,018,659–210,632,459)	103,943,952 (47,018,659–210,632,459)	NA†	5,450 (2,194–17,127)	5,450 (2,194–17,127)
Other	NA†	443,359,823 (265,444,093–829,321,063)	443,359,823 (265,444,093–829,321,063)	NA†	213,538 (140,116–295,335)	213,538 (140,116–295,335)
Unknown aetiology	743,156,302 (556,385,124–936,441,593)	1,457,422,180 (940,738,945–2,433,895,574)	2,210,028,002 (1,647,325,833–3,181,283,835)	17,326 (8,160–27,175)	741,975 (616,679–887,475)	759,397 (634,023–904,803)
Total	863,503,727 (666,975,325–1,067,418,321)	3,717,513,213 (2,677,563,116–5,613,998,807)	4,584,413,427 (3,525,036,414–6,468,858,881)	24,199 (14,785–34,140)	1,560,985 (1,322,986–1,834,156)	1,585,227 (1,345,254–1,857,476)

^National studies approach applied to all countries in EURO and low mortality (subregion A) countries in AMRO and WPRO.

^^ Modified CHERG approach applied to all middle/high mortality countries in AFRO, AMRO, EMRO, SEARO and WPRO.

*Diarrhoeal *Campylobacter* only (GBS-*Campylobacter* cases and deaths not included).

**Diarrhoeal norovirus only (vomiting only norovirus cases not included).

***Diarrhoeal non-typhoidal *Salmonella* only (invasive non-typhoidal *Salmonella* cases and deaths not included).

†Estimates not available from national studies.

‡Assumed not to cause fatalities in EURO and low mortality countries.

^μ^Assumed not to cause fatalities.

**Fig 4:**
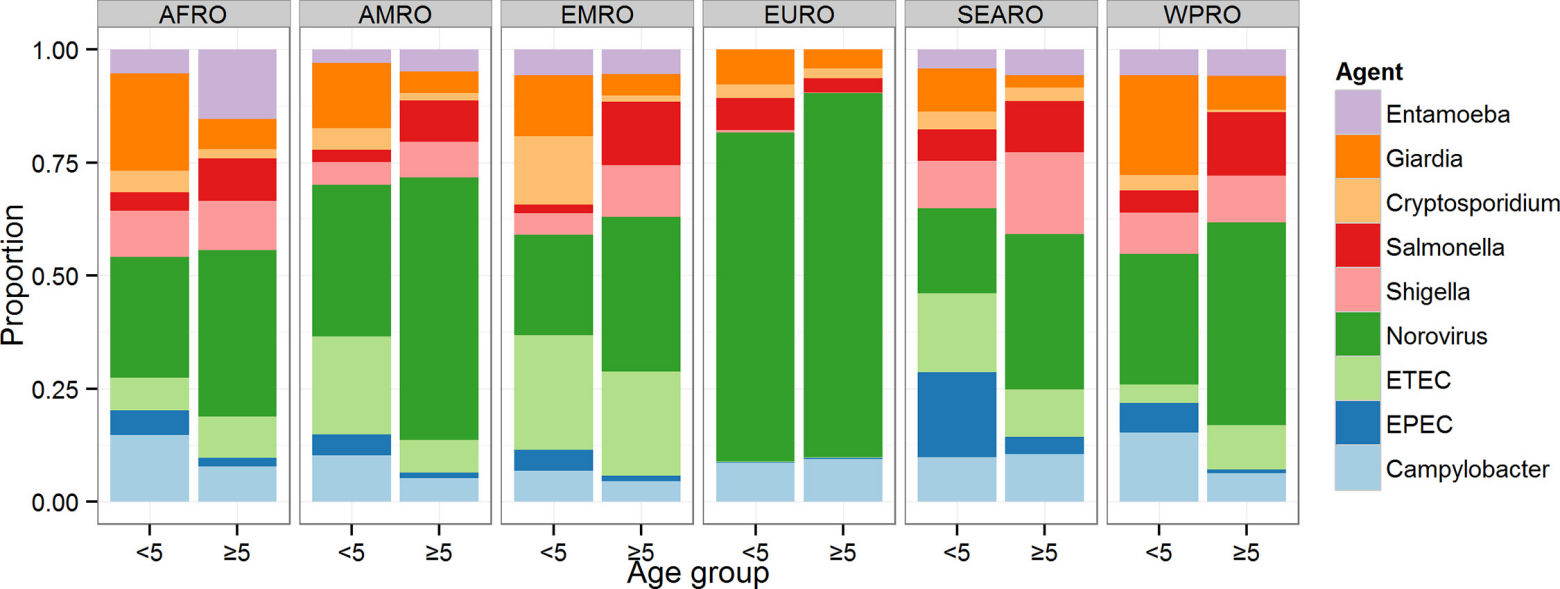
Relative contribution of nine diarrhoeal agents and of unknown aetiology for diarrhoeal cases in children <5 and the population ≥5 years of age. Estimates exclude the proportion of the incidence envelope attributable to “other pathogens”, because these were not considered in Approach 1 (applied to 60 countries within EURO, AMRO sub-region A, and WPRO sub-region A).

**Fig 5:**
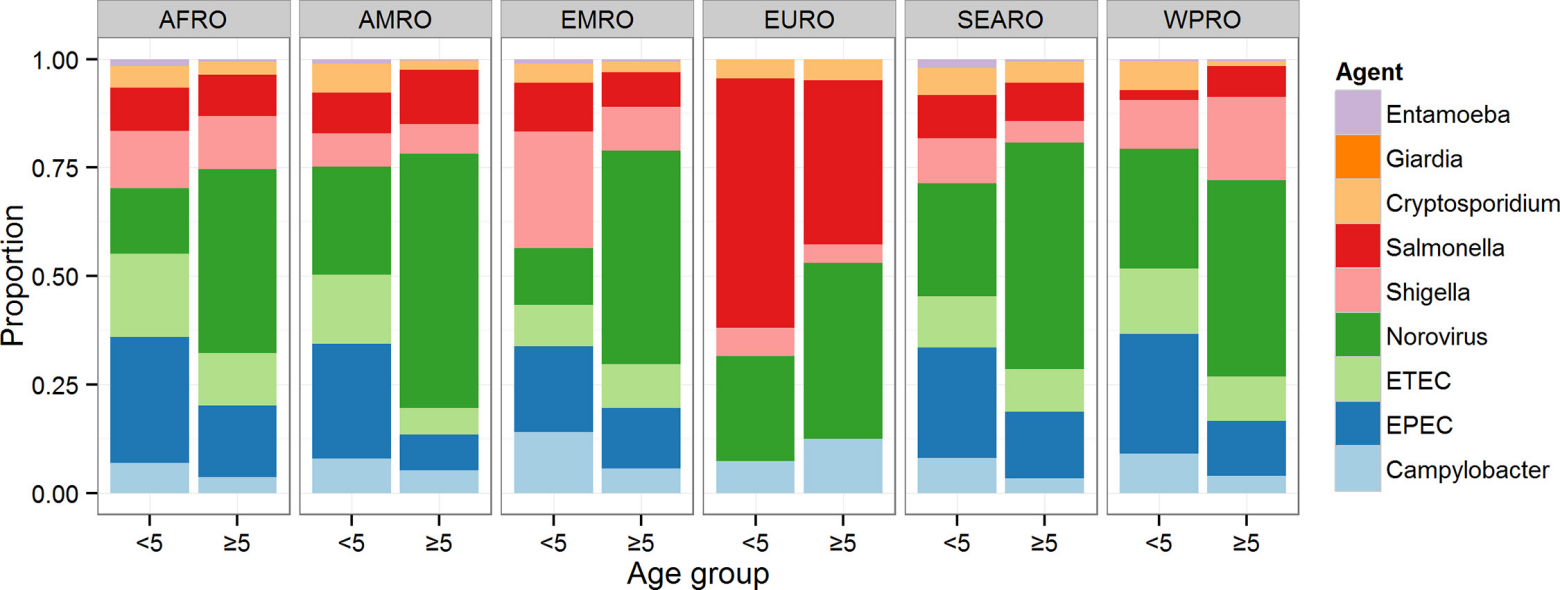
Relative contribution of nine diarrhoeal agents and of unknown aetiology for diarrhoeal deaths in children <5 and the population ≥5 years of age. Estimates exclude the proportion of the mortality envelope attributable to “other pathogens”, because these were not considered in Approach 1 (applied to 60 countries within EURO, AMRO sub-region A, and WPRO sub-region A).

There were marked regional differences in incidence and mortality caused by the nine diseases (Table 3; Figs 4 and 5). Among the nine pathogens, norovirus was the most common cause of cases in all regions, particularly among persons ≥5 years of age (Fig 4). Among these nine pathogens, norovirus and *G*. *lamblia* were the cause of the highest incidence in children <5 years of age; in AFRO, AMRO, EMRO and WPRO *Campylobacter* caused the third highest incidence in AFRO and WPRO, and *Cryptosporidium* spp. made a substantial contribution in EMRO. The second highest incidence of cases among persons ≥5 years of age was caused by *Salmonella* spp. in EMRO, SEARO and WPRO.

**Table 3:** Median rate per 100,000 of diarrhoeal illnesses and deaths by region, with 95% uncertainty intervals.

PATHOGEN	AFRO		AMRO		EMRO		EURO		SEARO		WPRO		GLOBAL	
	46 countries (847,062,195 pop.)		35 countries (937,213,444 pop.)		22 countries (589,012,258 pop.)		53 countries (898,751,529 pop.)		11 countries (1,789,987,553 pop.)		27 countries (1,818,793,708 pop.)			
	ILLNESSES (95% UI)	DEATHS (95% UI)	ILLNESSES (95% UI)	DEATHS (95% UI)	ILLNESSES (95% UI)	DEATHS (95% UI)	ILLNESSES (95% UI)	DEATHS (95% UI)	ILLNESSES (95% UI)	DEATHS (95% UI)	ILLNESSES (95% UI)	DEATHS (95% UI)	ILLNESSES (95% UI)	DEATHS (95% UI)
*Campylobacter*	3,987 (605–14,590)	1 (0.9–2)	2,099 (763–4,671)	0.09 (0.05–0.2)	2,871 (785–8,419)	1 (1–2)	755 (601–926)	0.05 (0.02–0.1)	2,380 (636–6,015)	0.7 (0.4–2)	1,512 (876–6,505)	0.06 (0.02–0.2)	2,415 (1,340–4,373)	0.5 (0.4–0.8)
*Cryptosporidium*	1,341 (392–3,616)	1 (0.6–2)	862 (498–1,605)	0.05 (0.03–0.1)	3,572 (1,928–6,236)	0.4 (0.1–1)	175 (69–380)	0.02 (0.006–0.05)	706 (251–2,551)	0.8 (0.5–2)	311 (138–633)	0.03 (0.006–0.1)	930 (626–1,521)	0.4 (0.3–0.6)
*Entamoeba histolytica*	2,807 (461–10,427)	0.2 (0.05–1)	1,069 (150–3,756)	0.006 (0.002–0.03)	2,947 (566–9,110)	0.06 (0.01–0.5)	0 (0–0)	0 (0–0)	1,119 (238–3,461)	0.1 (0.04–0.6)	1,033 (95–4,534)	0.005 (0.003–0.007)	1,511 (683–3,061)	0.08 (0.03–0.2)
EPEC	1,573 (637–3,324)	6 (4–8)	678 (190–2,022)	0.2 (0.1–0.4)	1,390 (503–3,199)	2 (1–4)	15 (7–26)	0 (0–0)	2,190 (340–7,191)	3 (2–4)	555 (351–835)	0.2 (0.1–0.3)	1,178 (592–2,491)	2 (1–2)
ETEC	2,970 (1,159–6,256)	4 (3–6)	3,816 (1,235–7,892)	0.1 (0.07–0.3)	14,201 (8,044–25,380)	1 (0.5–2)	15 (7–26)	0 (0–0)	3,016 (1,230–8,069)	2 (1–3)	1,559 (476–5,551)	0.1 (0.06–0.2)	3,501 (2,338–5,486)	1 (0.8–1)
*Giardia*	6,324 (3,364–11,273)	0 (0–0)	2,519 (957–5,288)	0 (0–0)	4,588 (2,374–8,211)	0 (0–0)	373 (297–454)	0 (0–0)	1,205 (246–3,773)	0 (0–0)	2,543 (1,337–5,108)	0 (0–0)	2,672 (1,890–3,820)	0 (0–0)
Norovirus	11,268 (5,943–22,906)	7 (4–10)	14,906 (10,063–24,036)	0.8 (0.6–1)	18,203 (10,391–31,219)	3 (2–4)	6,408 (4,858–8,059)	0.2 (0.1–0.2)	5,343 (1,550–23,924)	7 (5–10)	8,320 (3,858–21,440)	0.3 (0.2–0.5)	9,953 (7,135–16,320)	3 (2–4)
*Salmonella*	2,043 (448–6,146)	2 (2–3)	2,018 (1,118–3,578)	0.2 (0.1–0.3)	3,445 (336–27,248)	1 (1–2)	282 (193–391)	0.2 (0.1–0.3)	1,828 (239–8,499)	1 (0.8–3)	1,646 (346–10,731)	0.04 (0.03–0.05)	2,225 (941–5,555)	0.8 (0.6–1)
*Shigella*	3,407 (463–12,047)	3 (2–5)	1,926 (524–5,239)	0.1 (0.07–0.2)	4,143 (854–19,877)	3 (2–4)	23 (16–31)	0.02 (0.004–0.06)	3,343 (924–9,493)	1 (0.4–2)	2,038 (728–5,779)	0.1 (0.05–0.2)	2,774 (1,422–5,289)	1 (0.7–1)
Other diarrhoeal agents*	9,036 (2,936–22,252)	13 (7–20)	6,390 (2,950–12,386)	0.3 (0.2–0.5)	8,387 (2,160–24,151)	5 (3–8)	0 (0–0)	0 (0–0)	6,099 (1,130–22,628)	3 (0.7–6)	6,308 (2,771–16,592)	0.5 (0.4–0.7)	6,443 (3,858–12,053)	3 (2–4)

* Shiga toxin-producing *E*. *coli* and *Vibrio cholerae*.

For deaths caused by the nine pathogens, EPEC and norovirus were the most frequent causes in children <5 years of age in AFRO, AMRO, SEARO and WPRO (Fig 5). ETEC was also estimated to be an important cause of mortality, particularly in AFRO and AMRO, and *Shigella* spp. was the leading cause in EMRO and third leading in AFRO. *Salmonella* spp. caused the largest proportion of diarrhoeal deaths in this age group in EURO. In persons ≥5 years of age, norovirus was the leading cause of mortality in all regions.

## Discussion

These are the first global and regional estimates of incidence and mortality caused by nine pathogens that cause diarrhoea and are commonly transmitted through foods. Of these agents, we showed that norovirus was responsible for the largest number of cases followed by ETEC, *G*. *lamblia* and *Shigella* spp. Norovirus caused the most deaths among these nine pathogens, followed by EPEC and *Shigella* spp. Nearly 40% of all cases and 43% of deaths caused by these nine pathogens occurred in children <5 years of age, who represent only 8% of the global population. We also demonstrated regional differences in both the incidence and mortality of diseases caused by these nine pathogens. These differences are important to consider when defining interventions to reduce the burden of diseases commonly transmitted through foods. As an example, *Campylobacter* and *Salmonella*, which are pathogens that have mainly domestic food-producing animals as reservoirs and usually infect humans due to contamination in the food production chain, play a major role in the incidence of foodborne enteric disease in the EURO region but are less frequently observed in WHO-defined high mortality countries. In high mortality countries where poor sanitary conditions and food and water contamination are more important, pathogens such as *G*. *lamblia* and ETEC play more important roles. The predominance and relative ubiquity of norovirus across all regions suggests that targeted interventions, such as vaccines, may be necessary to reduce the burden of this human reservoir pathogen.

Our study focused only on diarrhoeal pathogens that are commonly transmitted through foods. It is important to recognize that the estimates we present include disease from all modes of transmission, including contaminated food, water, and environments, along with infected persons and animals. Our inclusion of only pathogens that are commonly transmitted through food also meant that we did not present estimates for important diarrhoeal agents, such as rotavirus. By including them into *other pathogens*, we have assured that we did not overestimate the incidence and mortality of remaining pathogens when distributing the diarrhoeal total cases and deaths to the nine diseases of interest.

We used two approaches to estimate the relative contribution of different aetiologies for disease because the quality and availability of data varied substantially between countries and regions. National incidence and mortality estimates were available only from seven low mortality countries, but given the high quality of these estimates, we gave priority to these data by using them as the basis for estimating the incidence and mortality of the diseases caused by the nine pathogens for all EURO and other low mortality countries (sub-region A in AMRO and WPRO). For all other countries, we adapted the CHERG approach for estimating the burden of diarrhoeal diseases in these countries [5]. This approach was facilitated by the availability of estimates of the envelope of diarrhoeal deaths, along with recent advances in diarrhoeal disease diagnosis, such as widespread application of polymerase chain reaction (PCR) for norovirus detection. We have divided countries for the two approaches on the basis of their overall mortality status, as defined by WHO, which we assume can be used as a proxy for differences in the public health status of the countries. The two approaches differed in terms of methodology, assumptions and type of data used. While approach 1 analysed national incidence and mortality of disease by pathogens commonly transmitted through foods estimated primarily by correcting surveillance data to account for underreporting and under-diagnosis, approach 2 relied on systematic reviews of studies identifying causative agents in patients with diarrhoea. Applying approach 2 for all regions was not possible due to the scarcity of community, inpatient and outpatient studies from low mortality countries for most pathogens. Because of these differences, we acknowledge that comparisons between regions should be made with care.

We relied on studies that estimated the incidence of potentially foodborne diseases in a specific country by correcting public health surveillance data for underreporting and under-diagnosis for Approach 1. Such studies have their own limitations, such as relying on a variety of data with variable quality and representativeness, multiple modelling approaches and a wide range of assumptions. As an example, most use population surveys on care-seeking behaviours of patients with diarrhoea, which are subject to recall bias that can influence the results [18]. Furthermore, in approach 1 we have assumed that the median aetiology-proportion of the seven collected studies (along with its uncertainty interval) represented the relative contribution of each agent for the diarrhoeal morbidity and mortality estimates, and have taken a simplified approach to estimate uncertainty.

Approach 2 to estimate the contribution of aetiologies for diarrhoeal cases and deaths used studies that tested for varying numbers of pathogens. Studies that focused on a single pathogen may tend to overestimate the importance of that pathogen because they are potentially more likely to be conducted in a study site with a high prevalence of that pathogen [5] or have selective inclusion criteria e.g. acute watery diarrhoea. However, we needed to include them due to the low number of studies focusing on multiple pathogens, particularly in the older age group. Due to scarcity of data, some regional aetiological proportion estimates were informed by a single study. To avoid that studies with potentially over or underestimated aetiology-proportion estimates misrepresented the estimate for that region, we have defined criteria to identify outliers, and have excluded these estimates from the results. These overestimations or underestimations can have various causes, such as study locations with a particularly high or low prevalence of that pathogen, a small sample size, or a country not representative of the whole region. Excluded outliers were treated as missing values, and the aetiology-global median used to represent the regional estimate. In this approach, we also assumed that the distribution of pathogens among inpatients hospitalised with severe diarrhoea represented the relative importance of these pathogens for diarrhoeal deaths. This meant that we used severity of disease as a proxy for mortality, which may lead to an over- or under-estimation of the number of deaths caused by some pathogens. In the analyses for persons over five years of age, due to sparseness of data we decided to use data from inpatient studies to estimate aetiology-proportion of cases, which may also have led to an erroneous estimation of the contribution of pathogens to diarrhoeal incidence. Additionally, this approach relied on stool samples, but some patients that tested positive for a pathogen may have had asymptomatic infections. This is more likely to happen for pathogens that have long excretion periods after illness (e.g. norovirus). Carefully conducted longitudinal studies would be needed to distinguish clinical cases from asymptomatic infections [5].

In approach 2, we estimated the proportion of the diarrhoeal cases and deaths that was caused by unknown aetiology. The defined strategy was to base our estimates on studies that had collected data for eight or more pathogens and had reported “aetiology unknown”. Undiagnosed cases could be caused by truly unrecognized agents, but also could be due to e.g. the use of incorrect or insensitive testing methods, to antimicrobial-therapy prior to stool sampling, or to non-infectious diarrhoeal causes. This strategy could only be adopted for the analyses for children <5 years of age, and still there were few studies available for the calculations. In remaining analyses, unknown was calculated as the difference between 100% and the sum of all remaining aetiologies (including *others*).

In both approaches, we assumed that studies conducted in a given country would be representative of the region or sub-region of this country. When several studies providing data for a given pathogen were available for that region, calculating a median estimate ensured that the influence of extreme values was restricted. On the contrary, when only one or few studies were available, this study’s/studies’ estimate was assumed to represent all countries within a region. In addition, the six regions’ classification was based on the geographic distribution of countries, and countries within each are very diverse. We tried to avoid inappropriate extrapolations by applying distinct approaches to low mortality and medium and high mortality countries, assuming that a country’s mortality status may also reflect the nation’s public health and food safety situation. In the EURO region, we made an extra extrapolation and assumed that all countries were similar to sub-region A countries in terms of foodborne diseases’ incidence and mortality. This region includes countries with other mortality status (EURO B and EURO C), and some of these countries may have a different distribution of aetiologies. Due to lack of data, we were not able to explore the extent of these differences.

A recent large prospective matched case-control study in children <5 years of age conducted over three years at selected sites in Africa and Asia (the GEMS study) estimated the burden of aetiology-specific diarrhoea in these sites, and concluded that rotavirus, *Cryptosporidium spp*., ETEC and *Shigella* spp. were the most important causes [7]. This study was fundamentally different from ours: among other differences, it calculated attributable cases of diarrhoea to each aetiology, comparing patients (moderate to severe diarrhoeal cases seen in health facilities) and controls (un-matched healthy children in a community sample). The GEMS study excluded mild diarrhoeal cases, which along with their moderate diarrhoeal cases would constitute our non-fatal incident cases. By including moderate and severe cases instead of only severe hospitalized cases, the GEMS study does not have a good proxy for fatal cases as we defined it. Still, the study’s findings are generally consistent with ours; EPEC, ETEC, *Shigella spp*. and *Cryptosporidium spp*. were among the most important causes of diarrhoea. One important difference was on the relative contribution of norovirus for diarrhoea incidence and mortality: our results suggest that norovirus is amongst the most important causes of diarrhoeal cases and deaths, whereas the GEMS study estimated a lower contribution of this pathogen. This discrepancy may indicate that our approach may lead to an overestimation of the proportion of diarrhoea attributable to norovirus or other pathogens that are likely to be carried asymptomatically after the first exposure (like certain types of ETEC, e.g. LT-ETEC) because it does not account for the potential detection of norovirus in the stool of healthy individuals [17]. However, the GEMS study required that control individuals did not have a diarrhoeal episode in the 7 days prior to sampling; because norovirus patients can excrete the virus for 3 to 4 weeks after infection, it is likely that some controls included in this study corresponded to asymptomatic carriers of norovirus [5]. These findings may suggest limitations in ascribing pathogenicity based on case-control studies, particularly in developing country settings in which frequent exposures and a high degree of transmission results in common reinfections [19]. A recent community based prospective cohort study (the “Etiology, Risk Factors, and Interactions of Enteric Infections and Malnutrition and the Consequences for Child Health and Development Project”, the MAL-ED study), supported our results and estimated that norovirus was the most important cause of diarrhoea in young children [20].

Our study provides information on the incidence and mortality of nine pathogens that cause diarrhoeal and are commonly transmitted through food. Most of these agents can also infect humans through other sources (e.g. environmental, contact with animals and person-to-person transmission), and the relative contribution of these sources for disease varies between aetiologies and regions [21]. To estimate the number of foodborne aetiology-specific diarrhoeal cases and deaths, our results need to be combined with regional source attribution-proportions and other aetiological agents [22]. These results, as well as the foodborne disease burden of each disease in terms of disability adjusted life years (DALYs) are presented elsewhere [23].

Our results provide public health policy makers, including risk managers, and other stakeholders with information for advocacy for improved regulation and control of diseases commonly transmitted through foods. We highlight the most important diarrhoeal diseases in different regions and age groups, which will allow policy makers to define and improve control strategies targeted at different pathogens, settings and countries.

## Supporting Information

S1 Appendix Estimating the aetiology-specific incidence and mortality of diarrhoea in 60 countries in the Region of the Americas (AMRO) sub-region A, Western Pacific Region (WPRO) sub-region A, and European Region (EURO; sub-regions A, B and C). (DOCX)

S2 Appendix Detailed description of the methods of the systematic reviews used to identify studies that provided data to derive aetiology-proportion estimates for all included pathogens except norovirus. (DOCX)

S3 Appendix PRISMA checklist. (PDF)

S1 File Contains supporting Tables A and B1-B4. Table A: Diarrhoeal cases and deaths in 2010 by age group and region for remaining 133 countries.†. Table B1: Regional and global etiology-proportions of diarrhea cases in children <5, 2010 (median % and 95% CI). Table B2: Regional and global etiology-proportions of diarrhea cases in persons >5, 2010 (median % and 95% CI). Table B3: Regional and global etiology-proportions of diarrhea deaths in children <5, 2010 (median % and 95% CI). Table B4: Regional and global etiology-proportions of diarrhea deaths in persons >5, 2010 (median % and 95% CI).
(DOCX)

S1 Table Total global incidence and mortality rate (per 100,000) of 10 foodborne diarrhoeal pathogens (median and 95% Uncertainty Intervals). (DOCX)
