# Supplementary material for: Aetiology-Specific Estimates of the Global and Regional Incidence and Mortality of Diarrhoeal Diseases Commonly Transmitted through Food
Source: PLoS One. 2015 Dec 3;10(12):e0142927. doi: 10.1371/journal.pone.0142927 (PMC4668836; doi:10.1371/journal.pone.0142927)
Supplement: S1 Appendix — (DOCX) [file pone.0142927.s001.docx]

**S1 Appendix. Estimating the aetiology-specific incidence and mortality of diarrhoea in 60 countries in the WHO’s Region of the Americas (AMRO) sub-region A, Western Pacific Region (WPRO) sub-region A, and European Region (EURO; sub-regions A, B and C)**

We estimated the incidence of infections and deaths due to nine diarrheal diseases (caused by diarrheal pathogens [non-typhoidal *Salmonella*, *Shigella spp.*, norovirus, enterotoxigenic *Escherichia coli* (ETEC), enteropathogenic *Escherichia coli* (ETEC), *Campylobacter spp.*, *Giardia lamblia, Entamoeba histolytica,* Cryptosporidium spp.) and due to diarrheal disease caused by unknown diarrheal agents in 60 countries in the WHO’s Region of the Americas (AMRO) sub-region A, Western Pacific Region (WPRO) sub-region A, and European Region (EURO; sub-regions A, B and C).

First, we conducted a literature review of national estimates of foodborne illnesses that included five or more of the nine diarrheal diseases. We identified national incidence estimates for seven countries: Australia, Canada, France, Netherlands, New Zealand, United States of America, and the United Kingdom [1-7]. Collected studies published incidence estimates or a range of incidence estimates, or mortality rate estimates or a range of mortality rate estimates for some or all pathogens. For studies that did not provide a mortality rate, we calculated it from the set of ranges from remaining studies. Some studies that only provided estimates of domestically-acquired infections (Australia [3] and Canada [6]): for these countries, we used the proportion of travel-related infections as estimated for New Zealand [1] and the United States [4] as proxies for the proportion of infections acquired during international travel in Australia and Canada, respectively. None of the studies provided incidence or mortality estimates for *Entamoeba histolytica.* Table S1.1. Presents all collected data on incidence and mortality of diarrhoeal diseases.

Table S1.1. Incidence and mortality of diarrhoeal diseases in seven countries within AMRO A, EURO and WPRO A. Estimates for marked countries are the result of transformations after correcting for total cases**.**

|  | **Country** | Australia^1**^ | Canada^2***^ | France^3^ | Netherlands^4^ | New Zealand^5^ | UK^6^ | USA^7^ |
| --- | --- | --- | --- | --- | --- | --- | --- | --- |
|  | **Year** | 2010 | 2006 | 2000 | 2009 | 2009 | 2009 | 2006 |
|  | **Population*** | 22,182,700 | 32,500,000 | 59,213,000 | 16,500,000 | 4,368,000 | 62,066,000 | 299,000,000 |
| ***Campylobacter*** | Incidence | 1128.72 | 789.23 | 31.79 | 557.58 | 8321.66 | 921.52 | 442.19 |
|  | Mortality | 0.014 |  | 0.026 | 0.23 | 0.046 |  | 0.04 |
| **EPEC** | Incidence |  |  |  |  |  |  | 13.33 |
|  | Mortality |  |  |  |  |  |  |  |
| **ETEC** | Incidence |  |  |  |  |  |  | 13.3 |
|  | Mortality |  |  |  |  |  |  |  |
| ***Salmonella*** | Incidence | 301.49 | 373.59 | 63.76 | 212.12 | 1067.26 | 62.2 | 411.04 |
|  | Mortality | 0.08 |  | 0.53 | 0.24 | 0.069 |  | 0.15 |
| ***Shigella*** | Incidence | 21.23 | 23.62 | 3.31 |  | 100.85 |  | 165.52 |
|  | Mortality |  |  |  |  |  |  | 0.013 |
| **Norovirus** | Incidence | 7057.3 | 10503.97 | 846.75 | 3781.82 | 12814.08 | 4680.95 | 6978.58 |
|  | Mortality | 0.0045 |  |  | 0.34 | 0.18 |  | 0.19 |
| ***Cryptosporidium*** | Incidence | 87.15 | 84.91 |  | 169.7 | 2019.18 | 70.62 | 250.21 |
|  | Mortality |  |  |  | 0.012 |  |  | 0.015 |
| ***Giardia lamblia*** | Incidence | 183.35 | 360.58 |  | 503.03 | 1712.8 | 84.48 | 408.55 |
|  | Mortality |  |  |  | 0.012 |  |  | 0.011 |
| **Unknown** | Incidence | 57,702.62 |  |  |  |  |  | 47,424.75 |
|  | Mortality |  |  |  |  |  |  | 1.19 |

*: Population data was given in the cited reference or from [http://esa.un.org/unpd/wpp/unpp/panel_population.htm for the year 2010](http://esa.un.org/unpd/wpp/unpp/panel_population.htm%20for%20the%20year%202010)

** Article reported domestically-acquired infections only.

*** Article reported domestically-acquired infections only.

As a second step, we have estimated the aetiology-specific median incidence and mortality of all studies that provided data for each pathogen, along with a 95% Confidence Interval (CI). These median estimates were then applied to the remaining 53 countries for which no data were available.

**References**

1. Cressey P, Lake R. *Estimated Incidence of Foodborne Illness in New Zealand: Application of Overseas Models and Multipliers*. Christchurch, New Zealand: Institute of Environmental Science and Research Limited; 2011.
2. Havelaar AH, Haagsma JA, Mangen MJ, et al. Disease burden of foodborne pathogens in the Netherlands, 2009. Int J Food Microbiol. 2012;156(3):231-8.
3. Kirk M, Ford L, Glass K, Hall G. *Changes in Foodborne Illness Acquired in Australia, circa 2000-circa 2010*. Australia: Australian Government; 2010. Submitted.
4. Scallan E, Hoekstra RM, Angulo FJ, Tauxe RV, Widdowson MA, Roy SL, Jones JL, Griffin PM. Foodborne illness acquired in the United States—major pathogens. Emerging Infect Dis. 2011;17(1):7-15.
5. Tam CC, Rodrigues LC, Viviani L, et al. Longitudinal study of infectious intestinal disease in the UK (IID2 study): incidence in the community and presenting to general practice. Gut. 2012;61(1):69-77.
6. Thomas MK, Murray R, Flockhart L, et al. Estimates of the burden of foodborne illness in Canada for 30 specified pathogens and unspecified agents, circa 2006. Foodborne Pathog Dis. 2013;10(7):639-48.
7. Vaillant V, De valk H, Baron E, et al. Foodborne infections in France. Foodborne Pathog Dis. 2005;2(3):221-32.
