# Supplementary material for: Aetiology-Specific Estimates of the Global and Regional Incidence and Mortality of Diarrhoeal Diseases Commonly Transmitted through Food
Source: PLoS One. 2015 Dec 3;10(12):e0142927. doi: 10.1371/journal.pone.0142927 (PMC4668836; doi:10.1371/journal.pone.0142927)
Supplement: S2 Appendix — (DOCX) [file pone.0142927.s002.docx]

**S 2 Appendix. Detailed description of the methods of the systematic reviews used to identify studies that provided data to derive aetiology-proportion estimates for all included pathogens except norovirus.**

We reviewed the evidence collected through four different systematic reviews to identify studies that provided data to estimate aetiology-proportions of diarrhoea cases and deaths. The original systematic reviews have been published (as described below). We then updated these reviews using the identical search strategy to include studies published in more recent years. All systematic reviews were conducted according to CHERG guidelines that have been described in detail elsewhere ([www.cherg.org](http://www.cherg.org)) [1, 2]. The reviews collected data on the following pathogens: Rotavirus, Escherichia coli (ETEC and EPEC), non-typhoidal Salmonella, Shigella, Campylobacter, Giardia lamblia, Vibrio cholerae, Cryptosporidium, Entamoeba histolytica, norovirus, astrovirus, adenovirus, and “unknown pathogen.

1. **Aetiology of diarrhoea deaths in children 0-4 years of age.** The details and results of this systematic review have been published elsewhere [3]. The review collected articles published between 1990 and 2011 reporting at least one of 13 diarrhoeal pathogens in children <5 years of age hospitalized with diarrhoea, and included 2011 rotavirus data from the Rotavirus Surveillance Network coordinated by WHO.

2. **Aetiology of diarrhoea cases in children 0-4 years of age.** This review was conducted in conjunction with the SR described in 1, but its results have not been published. It collected articles from the same time period that analysed stool samples from children < 5 years of age that had visited health facilities (outpatients) or that took part in community studies and reported at least of the 13 diarrhoeal pathogens.

**3. Aetiology of diarrhoea cases and deaths of diarrhoea in the population ≥5.** The details of this review have been published elsewhere [4]. The authors identified all papers reporting the proportion of diarrhoea episodes with positive laboratory tests for at least one pathogen in inpatient, outpatient and community settings through 1980 to 2008.

**4^[[1]](#footnote-1)^. Aetiology of diarrhoea cases and deaths in children 0-4 years and population ≥5: an update of previously conducted systematic reviews.** With the objective of updating the reviews described in 1 to 3, this review followed their methodologies to collect studies published from 2009 and 2012. Because the details and results of this review haven’t been published, we describe them here in more detail.

4.1. Methods

We followed the protocol of [3; 4], using the same search terms, data bases and inclusion and exclusion criteria. We searched PubMed/Medline, CAB abstracts, System for Information on Grey Literature in Europe (SIGLE), and all World Health Organization (WHO) Regional Databases for studies published from January 1, 2009 through December 31, 2013 using the following search and MeSH terms: ‘‘diarrhea’’ (or ‘‘diarrhoea’’), ‘‘gastroenteritis’’, ‘‘rotavirus’’, ‘‘E.coli’’ (or ‘‘Escherichia coli’’), ‘Salmonella’’ (not ‘‘typhi’’), ‘‘Shigella’’, ‘Campylobacter’’, ‘‘Giardia lamblia’’, ‘‘Vibrio’’, ‘‘Cryptosporidium’’, ‘‘Entamoeba’’, ‘‘norovirus’’, ‘‘calicivirus’’, ‘‘Norwalk agent’’, using ‘‘AND children’’ as a search restriction. An example of one of the search instructions in Medline PubMed is: ‘‘diarrhea’’[-mesh] OR ‘‘diarrhea’’[all fields] or ‘‘diarrhoea’’[all fields] OR ‘‘gastroenteritis’’[mesh] OR ‘‘gastroenteritis’’[all fields] OR ‘‘rotavirus’’[ mesh] OR ‘‘rotavirus’’[all fields] OR ‘‘E.coli’’[all fields] or ‘‘Escherichia coli’’[mesh] OR ‘‘Escherichia coli’’[all fields] OR

‘‘Salmonella’’[mesh] OR ‘‘Salmonella’’[all fields] OR ‘‘Shigella’’[- mesh] OR ‘‘Shigella’’[all fields] OR ‘‘Campylobacter’’[mesh] OR ‘‘Campylobacter’’[all fields] OR ‘‘Giardia lamblia’’[mesh] OR ‘‘Giardia lamblia’’[all fields] OR ‘‘Vibrio’’[mesh] OR ‘‘Vibrio’’[all fields] OR ‘‘Cryptosporidium’’[mesh] OR ‘‘Cryptosporidium’’[all fields] OR ‘‘Entamoeba’’[mesh] OR ‘‘Entamoeba’’[all fields] OR ‘‘norovirus’’[mesh] OR ‘‘norovirus’’[all fields] OR ‘‘calicivirus’’[ all fields] OR ‘‘Norwalk agent’’[all fields] AND ‘‘children’’[all fields]. The objective of the search was to identify all papers reporting the proportion of diarrhoea episodes with positive laboratory tests for at least one pathogen in in-patient, out-patient and community settings that met our inclusion and exclusion criteria.

We included studies published in all languages and conducted in children < 5 years, adolescents and adults with at least 12 months of surveillance to minimize bias due to seasonality of diarrhea pathogens. We excluded studies enrolling only patients with clinical signs of dysentery, i.e. blood in the stool, studies conducted in special populations such as travelers, patients hospitalized for other reasons, or only HIV positive persons and all individual or outbreak case reports. Studies that did not screen for HIV status and/or did not enroll based on HIV status were included. All exclusion criteria were chosen to ensure study populations represented the general population in the study community.

We extracted data on all enteric pathogens investigated in included studies. All data were compiled in a standardized data base in Excel. We then selected data on the pathogens of interest for our study, and excluded all studies that focused on norovirus only.

4.2. Results

The search for studies for the overall population (including children and adults) identified 2,610 studies with possible etiology data (Figure SI2.1). Of these, 145 abstracts were reviewed for eligibility, and 142 papers screened for possible etiology data. 22 studies met the inclusion and exclusion criteria. These studies included community studies, outpatient studies, inpatient studies and studies without identified setting (classified as *unknown*). Forty seven additional papers met the inclusion criteria, but were excluded because they lacked information; of these, eight studies were excluded because they lacked information on the sample size.


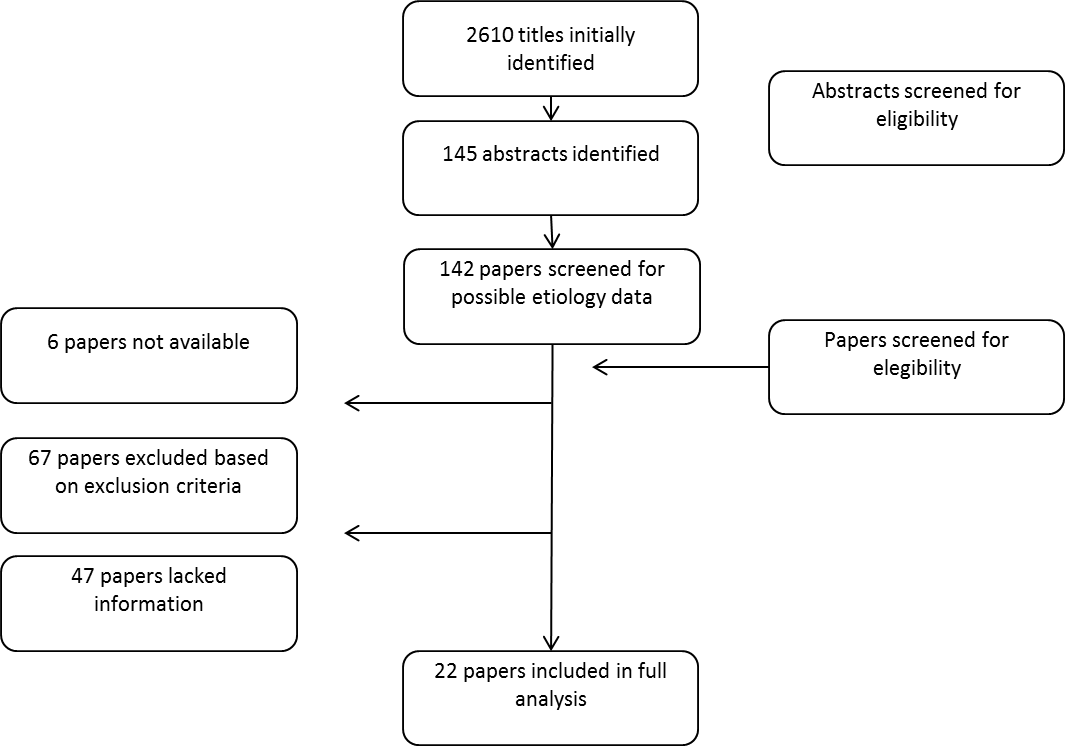


Fig SI2.1. Results of systematic literature review: studies collecting data for the population ≥5. The 22 papers included in the full analysis correspond to references [5 - 26]

The selection process for the articles focused on children <5 years of age differed on the eligibility criteria, and thus results are presented separately (Figure SI2.2). Of 25 papers screened for possible etiology data, 11 studies were selected for analysis. To these, we added information for this age category extracted from 10 studies collected through the first search.


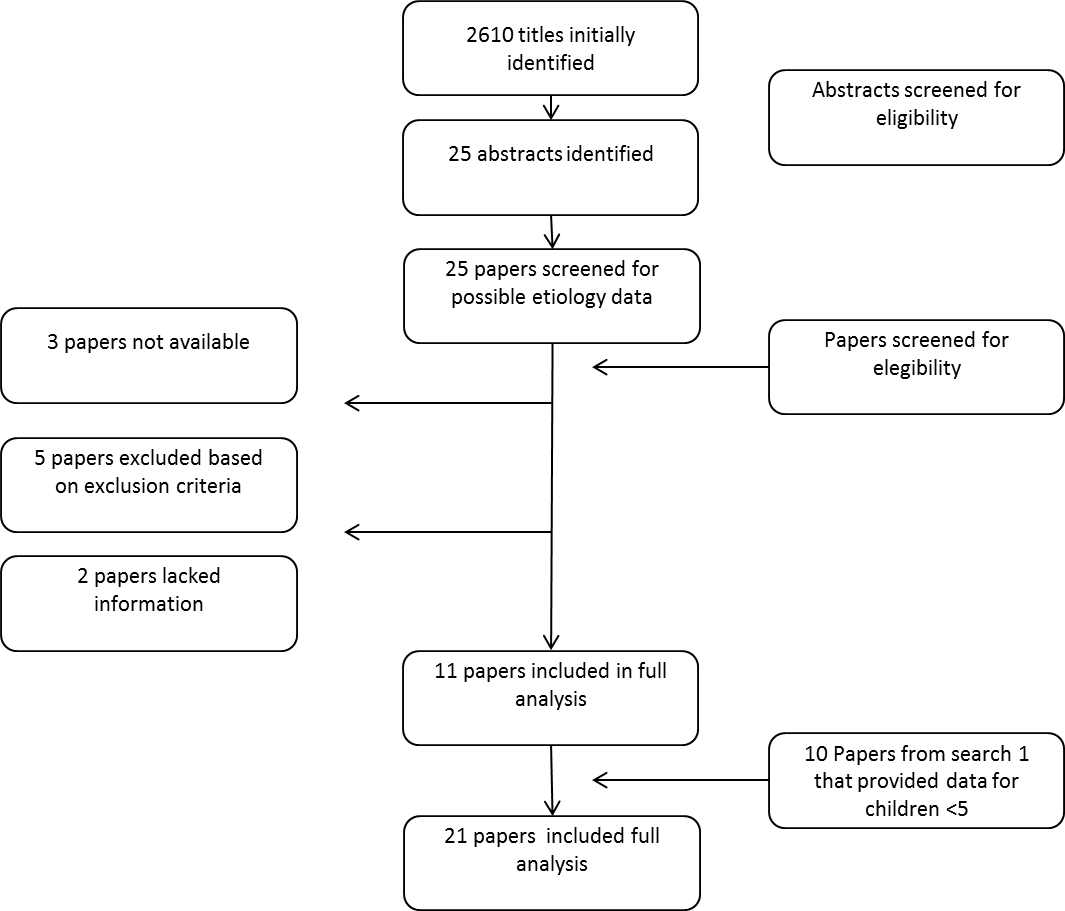


Fig SI2.2. Results of systematic literature review for etiology of diarrhea in children <5. The 11 papers included in full analysis that were not in the first search correspond to references [27-37]

The collected studies provided data on 29 agents: EIEC, ETEC, EPEC, EAEC, CTEC, STEC, unspecified E. coli, Salmonella spp, Shigella spp., Campylobacter spp., Yersinia spp., Adenovirus, Rotavirus, Sapovirus, Astrovirus, Bocavirus, Giardia spp., Calicivirus/Norovirus/Norwalk, Cryptosporidium, E. histolytica, V. cholera, V. paraheamolyticus, C. difficile, B. hominis, B. cereus, C. perfringens, L. monocytogenes, S. aureus, and “unspecified agents”. Of these, studies that did not provide data for any of the agents of interest for FERG or that collected data for norovirus only were excluded from the final dataset for analysis. We excluded four studies from the final dataset for the population ≥5, and 14 studies from the dataset for children <5.

**References**

1. Rudan I, Lawn J, Cousens S, Rowe AK, Boschi-Pinto C, Tomaskovic L, et al. Gaps in policy-relevant information on burden of disease in children: a systematic review. Lancet. 2005; 365(9476): 2031-40.
2. Walker N, Fischer-Walker C, Bryce J, Bahl R, Cousens S. Standards for CHERG reviews of intervention effects on child survival. Int J Epidemiol. 2010; 39 Suppl 1: i21-31.
3. Lanata CF, Fischer-Walker CL, Olascoaga AC, Torres CX, Aryee MJ, Black RE, et al. Global causes of diarrheal disease mortality in children <5 years of age: a systematic review. PloS one. 2013;8(9):e72788. PubMed PMID: 24023773. Pubmed Central PMCID: 3762858.
4. **Fischer Walker CL,** Sack D, Black RE. Etiology of diarrhea among older children, adolescents, and adults: a systematic review.  PLoS Negl Trop Dis 2010; 4(8): e768.
5. Alikhani MY, Sedighi I, Zamani A, Aslani MM, Sadrosadat T. Incidence of diarrhoeagenic escherichia coli isolated from young children with diarrhoea in the west of iran. *Acta Microbiol Immunol Hung*. 2012;59(3):367-374.
6. Cheun H, Cho S, Lee J, et al. Infection status of hospitalized diarrheal patients with gastrointestinal protozoa, bacteria, and viruses in the republic of korea. *Korean J Parasitol*. 2010;48(2):113-120.
7. Chowdhury F, Rahman MA, Begum YA, et al. Impact of rapid urbanization on the rates of infection by vibrio cholerae O1 and enterotoxigenic escherichia coli in dhaka, bangladesh. *PloS Neglected Tropical Diseases*. 2011;5(4).
8. Hasing ME, Trueba G, Baquero MI, Ponce K, Cevallos W, Solberg OD, Eisenberg JN. Rapid changes in rotaviral genotypes in Ecuador. J Med Virol. 2009;81(12):2109-13
9. Khananurak K, Vutithanachot V, Simakachorn N, Theamboonlers A, Chongsrisawat V, Poovorawan Y. Prevalence and phylogenetic analysis of rotavirus genotypes in thailand between 2007 and 2009. *Infection, Genetics and Evolution*. 2010;10(4):537-545.
10. Lopman BA, Hall AJ, Curns AT, Parashar UD. Increasing rates of gastroenteritis hospital discharges in US adults and the contribution of norovirus, 1996-2007. *Clinical Infectious Diseases*. 2011;52(4):466-474.
11. Paul SK, Ahmed MU, Hossain MA, Mahmud MC, Bhuiyan MR, Saha SK, Tabassum S. Molecular characterization of group A human rotavirus among hospitalized children and adults in Bangladesh: Finding of emerging GI2 strain. Mymensingh Med J. 2010;19(1):16-26.
12. Podkolzin AT, Fenske EB, Abramycheva NY, Shipulin GA, Sagalova OI, Mazepa VN, Ivanova GN, Semena AV, Tagirova ZG, Alekseeva MN, Molochny VP, Parashar UD, Vinjé J, Maleev VV, Glass RI, Pokrovsky VI. Hospital-based surveillance of rotavirus and other viral agents of diarrhea in children and adults in Russia, 2005–2007. J Infect Dis. 2009;200(1):228-33.
13. Qu M, Deng Y, Zhang X, et al. Etiology of acute diarrhea due to enteropathogenic bacteria in beijing, china. *J Infect*. 2012;65(3):214-222.
14. Rajabally N, Pentecost M, Pretorius G, Whitelaw A, Mendelson M, Watermeyer G. The clostridium difficile problem: A south african tertiary institution's prospective perspective. *South African Medical Journal.* 2013;103(3):168-172.
15. Soofi SB, Habib MA, von Seidlein L, Khan MJ, Muhammad S, Bhutto N, Khan MI, Rasool S, Zafar A, Clemens JD, Nizami Q, Bhutta ZA. A comparison of disease caused by Shigella and Campylobacter species: 24 months community based surveillance in 4 slums of Karachi, Pakistan. J Infect Public Health. 2011;4(1):12-21.
16. Tan DM, Liu W, Deng LL. Norovirus Infection in adults with sporadic gastroenteritis during 2007-2008 in Nanning municipal. Zhongguo Yi Miao He Mian Yi. 2010;16(2):132-5.
17. Wang F, Wang Y, Peng J, et al. Genetic characterization of human astrovirus infection in wuhan, people's republic of china, 2007-2008. *Can J Microbiol*. 2011;57(11):964-968.
18. Banerjee M, Nair GB, Ramamurthy T. Phenotypic & genetic characterization of bacillus cereus isolated from the acute diarrhoeal patients. *Indian J Med Res*. 2011;133(1):88-95.
19. Chow BDW, Ou Z, Esper FP. Newly recognized bocaviruses (HBoV, HBoV2) in children and adults with gastrointestinal illness in the united states. *Journal of Clinical Virology.* 2010;47(2):143-147.
20. Das S, Choudhry S, Saha R, Ramachandran VG, Kaur K, Sarkar BL. Emergence of multiple drug resistance vibrio cholerae O1 in east delhi. *The Journal of Infection in Developing Countries*. 2011;5(4):294-298.
21. Herwana E, Surjawidjaja JE, Salim OC, Indriani N, Bukitwetan P, Lesmana M. Shigella-associated diarrhea in children in south jakarta, indonesia. *Southeast Asian J Trop Med Public Health*. 2010;41(2):418-425.
22. Hilmarsdottir I, Baldvinsdottir GE, Haroardottir H, Briem H, Sigurosson SI. Enteropathogens in acute diarrhea: A general practice-based study in a nordic country. *European Journal of Clinical Microbiology and Infectious Diseases*. 2012;31(7):1501-1509.
23. Hinenoya A, Naigita A, Ninomiya K, et al. Prevalence and characteristics of cytolethal distending toxin-producing escherichia coli from children with diarrhea in japan. *Microbiol Immunol*. 2009;53(4):206-215.
24. Park D, Kim J, Park J, et al. Epidemiological analysis of norovirus infection between march 2007 and february 2010. *Korean Journal of Laboratory Medicine*. 2010;30(6):647-653.
25. Chen J, Sun XT, Zeng Z, Yu YY. Campylobacter enteritis in adult patients with acute diarrhea from 2005 to 2009 in Beijing, China. Chin Med J 2011; 124:1508-1512.
26. GiugnoS, OderizS. Bacterial etiology of acute diarrhea in pediatric patients. Acta Bioquím Clín Latinoam 2010; 44: 63-69.
27. Lou J, Xu X, Wu Y, Tao R, Tong M. Epidemiology and burden of rotavirus infection among children in hangzhou, china. *Journal of Clinical Virology*. 2011;50(1):84-87.
28. Jere KC, Sawyerr T, Seheri LM, et al. A first report on the characterization of rotavirus strains in sierra leone. *J Med Virol*. 2011;83(3):540-550.
29. Martin-Ampudia M, Mariscal A, Lopez-Gigosos R, Mora L, Fernandez-Crehuet J. Under-notification of cryptosporidiosis by routine clinical and laboratory practices among non-hospitalised children with acute diarrhoea in southern spain. *Infection*. 2012;40(2):113-119.
30. Sadeghian H, Sadeghian M, Sadeghian A, Hamedi A. Incidence of rotavirus diarrhea in children under 6 years referred to the pediatric emergency and clinic of ghaem hospital, mashhad, iran. *Acta Med Iran*. 2010;48(4):263-265.
31. Shoukry NM, Dawoud HA, Haridy FM. Studies on zoonotic cryptosporidiosis parvum in ismailia governorate, egypt. *J Egypt Soc Parasitol*. 2009;39(2):479-488.
32. Yang XL, He JH, Yang XT. Detection of rotavirus in 2745 children with diarrhea. *Chinese Journal of Contemporary Pediatrics*. 2011;13(3):255-257.
33. Zeng M, Gong ZX, Zhang YX, Zhu QR, Wang XH. Prevalence and genetic diversity of norovirus in outpatient children with acute diarrhea in shanghai, china. *Jpn J Infect Dis*. 2011;64(5):417-422.
34. Zeng M, Xu X, Zhu C, et al. Clinical and molecular epidemiology of norovirus infection in childhood diarrhea in china. *J Med Virol*. 2012;84(1):145-151.
35. Deng L, Jia LY, Qian Y, Chen DM, Zhang Y, Zhang YL. Comparative analysis on clinical manifestations for gastroenteritis caused by norovirus and rotavirus. Zhonghua Liu Xing Bing Xue Za Zhi. 2009 ;30:398-401.
36. Lorrot M, Bon F, El Hajje MJ, Aho S, Wolfer M, Giraudon H, Kaplon J, Marc E, Raymond J, Lebon P, Pothier P, Gendrel D. Epidemiology and clinical features of gastroenteritis in hospitalised children: prospective survey during a 2-year period in a Parisian hospital, France. Eur J Clin Microbiol Infect Dis. 2011 ;30:361-368.
37. Munford V, Gilio AE, de Souza EC, Cardoso DM, Cardoso Dd, Borges AM, Costa PS, Melgaço IA, Rosa H, Carvalho PR, Goldani MZ, Moreira ED Jr, Santana C, El Khoury A, Ikedo F, Rácz ML. Rotavirus gastroenteritis in children in 4 regions in Brazil: a hospital-based surveillance study. J Infect Dis. 2009 1;200 Suppl 1:S106-13.

1. This systematic review was commissioned by the Foodborne Diseases Epidemiology Reference Group (FERG), World Heath Organization [↑](#footnote-ref-1)
