# Supplementary material for: Aetiology-Specific Estimates of the Global and Regional Incidence and Mortality of Diarrhoeal Diseases Commonly Transmitted through Food
Source: PLoS One. 2015 Dec 3;10(12):e0142927. doi: 10.1371/journal.pone.0142927 (PMC4668836; doi:10.1371/journal.pone.0142927)
Supplement: S1 File — Table A: Diarrhoeal cases and deaths in 2010 by age group and region for remaining 133 countries.†. Table B1: Regional and global etiology-proportions of diarrhea cases in children <5, 2010 (median % and 95% CI). Table B2: Regional and global etiology-proportions of diarrhea cases in persons >5, 2010 (median % and 95% CI). Table B3: Regional and global etiology-proportions of diarrhea deaths in children <5, 2010 (median % and 95% CI). Table B4: Regional and global etiology-proportions of diarrhea deaths in persons >5, 2010 (median % and 95% CI). (DOCX) [file pone.0142927.s004.docx]

Table A: Diarrheal cases and deaths in 2010 by age group and WHO region for 133 low and middle-income countries.†.

|  | **Cases (Median and 95% uncertainty intervals (U))I*** | | **Deaths(Median and 95% uncertainty intervals (U))**** | |
| --- | --- | --- | --- | --- |
|  | **<5 years** | **≥5 years** | **<5years** | **≥5 years** |
| **AFRO** | 452,895,639 (277,700,618-692,035,218) | 246,548,806 (37,404,467-853,268,418) | 366,044 (310,241-430,105) | 296,818 (251,563-348,760) |
| **AMRO** | 171,228,102 (141,361,082-205,993,346) | 280,692,174 (141,445,476-536,156,624) | 8,055 (6,825-9,467) | 15,027 (12,736-17,657) |
| **EMRO** | 203,598,060 (125,980,141-309,139,921) | 430,019,467 (223,347,114-752,738,426) | 98,229 (83,252-115,417) | 44,238 (37,493-51,980) |
| **SEARO** | 419,381,313 (281,924,055-597,631,096) | 523,612,592 (86,143,054-1,734,530,091) | 196,528 (166,556-230,908) | 498,505 (422,499-585,742) |
| **WPRO** | 246,725,621 (189,166,565-316,442,523) | 603,430,422 (235,961,741-1,393,900,206) | 23,944 (20,294-28,135) | 13,605 (11,531-15,986) |
| **TOTAL** | 1,504,092,544 (1,232,550,361-1,833,517,669) | 2,195,792,033 (1,233,825,639-4,073,594,863) | 692,793 (587,165-814,031) | 868,192 (735,821-1,020,125) |

* Estimates from Fisher-Walker 2010 and 2013 (total envelopes).

** Estimates from WHO 2013 (total envelopes).

†EURO region, AMRO sub-region A, or WPRO sub-region A not included.

| Table B1. Regional and global etiology-proportions of diarrhea cases in children <5, 2010 (median % and 95% CI).* | | | | | | |
| --- | --- | --- | --- | --- | --- | --- |
| **Etiology** | **AFRO** | **AMRO** | **EMRO** | **SEARO** | **WPRO** | **GLOBAL**** |
| **Salmonella** | 1.38 | 1.30 | 1.09 | 2.18 | 2.33 | 2.17 |
|  | [0.05; 7.06] | [0.20; 4.31] | [0.65; 1.70] | [0.06; 11.45] | [1.43; 3.60] | [0.16; 7.19] |
| **Campylobacter** | 5.33 | 5.60 | 3.62 | 3.52 | 7.37 | 7.29 |
|  | [0.13; 23.59] | [0.68; 17.70] | [0.84; 9.35] | [0.29; 13.49] | [5.53; 9.59] | [3.62; 16.70] |
| **EPEC** | 2.45 | 2.38 | 2.43 | 6.71 | 3.14 | 3.90 |
|  | [1.01; 5.02] | [0.18; 9.58] | [0.42; 6.87] | [0.21; 26.5] | [2.02; 4.59] | [1.61; 11.78] |
| **ETEC** | 3.27 | 13.3 | 14.76 | 7.41 | 1.95 | 8.40 |
|  | [1.13; 7.13] | [2.43; 30.83] | [10.03; 20.46] | [3.43; 14.41] | [1.06; 3.23] | [2.90; 16.40] |
| **Shigella** | 3.63 | 2.36 | 2.67 | 3.95 | 4.10 | 4.54 |
|  | [0.10; 16.79] | [0.07; 11.51] | [1.30; 4.78] | [0.57; 12.64] | [1.17; 9.53] | [0.00; 14.43] |
| **Giardia** | 10.21 | 8.36 | 7.76 | 3.33 | 10.53 | 12.20 |
|  | [6.22; 15.38] | [1.30; 22.48] | [3.78; 13.36] | [0.25; 13.38] | [5.52; 16.85] | [0.87; 19.20] |
| **Cryptosporidium** | 2.06 | 2.96 | 8.75 | 1.67 | 1.48 | 3.04 |
|  | [0.53; 5.21] | [1.68; 4.96] | [5.53; 12.97] | [0.73; 3.48] | [0.42; 3.58] | [1.82; 5.97] |
| **Entamoeba** | 1.13 | 0.80 | 2.49 | 1.17 | 1.36 | 1.11 |
|  | [0.001; 12.58] | [0.0007; 9.69] | [0.11; 11.14] | [0.01; 7.43] | [0.002; 13.15] | [0.00; 18.55] |
| **Norovirus** | 12.83 | 20.00 | 12.98 | 7.96 | 13.49 | 19.00 |
|  | [9.79; 15.93] | [13.95; 27.85] | [9.49; 16.97] | [3.19; 16.67] | [8.65; 19.29] | [17.13; 20.04] |
| **Unknown** | 39.85 | 25.67 | 27.91 | 39.77 | 36.26 | 45.51 |
|  | [30.44; 49.42] | [18.71; 34.69] | [20.97; 35.42] | [17.01; 59.86] | [28.99; 43.33] | [37.94; 59.18] |
| **Others** | 11.36 | 9.84 | 12.44 | 14.72 | 15.51 | 16.67 |
|  | [3.17; 24.00] | [1.18; 26.20] | [2.53; 28.17] | [1.85; 36.26] | [11.21; 20.46] | [4.32; 49.58] |
| *Missing values (Norovirus in AMRO, EPEC and unknown in EMRO, and unknown in WPRO), and removed outliers (Salmonella in AMRO, and Giardia and Entamoeba in WPRO) were replaced by the global median etiology-proportions. | | | | | | |
| **Global medians estimated before a second proportion-adjustment to 100% (applied as part of the envelope-attribution step) | | | | | | |

| Table B2. Regional and global etiology-proportions of diarrhea cases in persons >5, 2010 (median % and 95% CI).* | | | | | | |
| --- | --- | --- | --- | --- | --- | --- |
| **Etiology** | **AFRO** | **AMRO** | **EMRO** | **SEARO** | **WPRO** | **GLOBAL**** |
| **Salmonella** | 3.64 | 5.24 | 4.25 | 3.79 | 4.12 | 3.76 |
|  | [2.06; 5.90] | [4.09; 6.62] | [0.01; 33.68] | [0.27; 15.65] | [0.08; 24.67] | [2.93; 15.67] |
| **Campylobacter** | 3.05 | 2.48 | 1.90 | 4.56 | 1.13 | 2.44 |
|  | [1.88; 4.55] | [0.60; 6.55] | [0.11; 8.31] | [4.35; 4.79] | [0.001; 13.42] | [0.99; 6.56] |
| **EPEC** | 0.69 | 0.68 | 0.69 | 1.62 | 0.36 | 0.68 |
|  | [0.25; 1.44] | [0.25; 1.46] | [0.25; 1.44] | [0.62; 3.43] | [0.25; 0.49] | [0.00; 1.40] |
| **ETEC** | 3.60 | 3.91 | 12.36 | 3.99 | 3.99 | 3.94 |
|  | [2.85; 4.46] | [0.77; 11.30] | [6.31; 21.18] | [0.70; 11.23] | [0.77; 11.47] | [2.78; 11.53] |
| **Shigella** | 3.54 | 4.23 | 4.43 | 7.69 | 4.32 | 4.22 |
|  | [0.42; 12.09] | [0.83; 12.00] | [0.11; 23.35] | [4.34; 12.23] | [1.12; 10.77] | [1.70; 12.12] |
| **Giardia** | 2.48 | 2.48 | 2.49 | 1.10 | 3.09 | 2.48 |
|  | [0.90; 5.27] | [0.93; 5.12] | [0.93; 5.14] | [0.36; 2.42] | [0.90; 7.15] | [0.33; 4.96] |
| **Cryptosporidium** | 0.61 | 0.61 | 0.60 | 0.97 | 0.24 | 0.59 |
|  | [0.04; 2.46] | [0.05; 2.52] | [0.05; 2.48] | [0.05; 4.57] | [0.11; 0.44] | [0.27; 2.51] |
| **Entamoeba** | 5.85 | 2.46 | 2.44 | 2.43 | 1.97 | 2.42 |
|  | [2.40; 11.40] | [0.26; 8.84] | [0.25; 8.97] | [1.27; 4.15] | [0.09; 9.33] | [0.59; 0.09] |
| **Norovirus** | 14.30 | 28.06 | 18.72 | 12.36 | 18.27 | 18.13 |
|  | [8.00; 22.55] | [19.00; 38.75] | [12.13; 26.93] | [1.01; 43.18] | [6.18; 36.67] | [5.91; 36.13] |
| **Unknown** | 51.38 | 32.49 | 40.16 | 48.07 | 43.30 | NA*** |
|  | [31.22; 65.18] | [15.38; 47.32] | [4.70; 59.96] | [7.04; 71.11] | [10.66; 66.41] |  |
| **Others** | 8.54 | 14.65 | 4.63 | 7.37 | 12.81 | 8.50 |
|  | [1.22; 25.87] | [7.77; 23.82] | [0.20; 22.5] | [0.11; 40.45] | [3.15; 31.17] | [2.32; 26.45] |
| *Missing values (Shigella , Giardia and Entamoeba in AMRO; Entamoeba in EMRO), and removed outliers (Cryptosporidium in AFRO; Campylobacter, EPEC, ETEC and Cryptosporidium in AMRO; Giardia and Cryptosporidium in EMRO; Salmonella in SEARO; Campylobacter and ETEC in WPRO) were replaced by the global median etiology-proportions. | | | | | | |
| **Global medians estimated before a second proportion-adjustment to 100% (applied as part of the envelope-attribution step) | | | | | | |
| ***NA: non-applicable. Calculated as the difference between the sum of all estimated etiology-proportions and 100%. | | | | | | |

| Table B3. Regional and global etiology-proportions of diarrhea deaths in children <5, 2010 (median % and 95% CI).* | | | | | | | | | | | | |
| --- | --- | --- | --- | --- | --- | --- | --- | --- | --- | --- | --- | --- |
| **Etiology** | **AFRO** | | **AMRO** | | **EMRO** | | **SEARO** | | **WPRO** | | **GLOBAL**** | |
| **Salmonella** | 3.07 | | 2.70 | | 6.05 | | 3.54 | | 0.74 | | 3.47 | |
|  | [1.81; 4.78] | | [0.19; 10.76] | | [4.66; 7.97] | | [0.06; 18.79] | | [0.56; 1.01] | | [0.81; 9.76] | |
| **Campylobacter** | 2.11 | | 3.61 | | 7.56 | | 3.51 | | 3.42 | | 4.20 | |
|  | [1.17; 3.52] | | [0.50; 11.81] | | [5.43; 10.47] | | [0.47; 11.42] | | [0.54; 10.28] | | [0.66; 12.88] | |
| **EPEC** | 8.95 | | 14.26 | | 10.46 | | 12.99 | | 11.75 | | 14.43 | |
|  | [5.36; 13.61] | | [3.65; 31.25] | | [4.42; 19.38] | | [8.95; 18.55] | | [6.35; 19.26] | | [7.10; 23.4] | |
| **ETEC** | 5.87 | | 7.78 | | 4.79 | | 5.25 | | 6.11 | | 7.56 | |
|  | [2.90; 10.17] | | [0.77; 24.29] | | [1.20; 12.04] | | [0.86; 15.3] | | [1.90; 13.86] | | [1.19; 17.25] | |
| **Shigella** | 3.96 | | 3.77 | | 14.54 | | 4.30 | | 4.31 | | 5.29 | |
|  | [1.45; 8.09] | | [1.25; 8.95] | | [10.33; 20.34] | | [0.30; 16.5] | | [0.85; 11.9] | | [0.10; 14.83] | |
| **Giardia** | NA*** | | NA | | NA | | NA | | NA | | NA | |
|  |  | |  | |  | |  | |  | |  | |
| **Cryptosporidium** | 1.48 | | 3.20 | | 1.90 | | 2.54 | | 2.27 | | 2.71 | |
|  | [0.58; 3.07] | | [0.66; 8.82] | | [0.15; 7.42] | | [0.23; 10.22] | | [0.19; 8.51] | | [0.00; 10.96] | |
| **Entamoeba** | 0.21 | | 0.29 | | 0.26 | | 0.53 | | 0.23 | | 0.29 | |
|  | [2.59; 3.06] | | [2.96; 1.07] | | [2.73; 4.56] | | [4.80; 7.93] | | [0.33; 2.61] | | [0.00; 3.87] | |
| **Norovirus** | 4.64 | | 11.81 | | 7.03 | | 13.12 | | 11.17 | | 13.01 | |
|  | [3.06; 6.66] | | [1.07; 33.87] | | [4.56; 10.53] | | [7.93; 20.55] | | [2.61; 24.72] | | [6.69; 15.61] | |
| **Unknown** | 41.15 | | 16.82 | | 15.71 | | 21.86 | | 18.12 | | NA**** | |
|  | [33.82; 49.74] | | [7.81; 30.39] | | [7.67; 26.14] | | [15.05; 31.13] | | [8.85; 29.83] | |  | |
| **Others** | 27.40 | | 28.78 | | 29.24 | | 26.63 | | 38.43 | | 22.39 | |
|  | [14.13; 39.75] | | [11.59; 47.86] | | [14.94; 42.80] | | [3.80; 49.19] | | [26.84; 51.15] | | [10.53; 39.47] | |
| *Missing values (Cryptosporium, Entamoeba and unknown in EMRO; EPEC, ETEC and unknown in WPRO), and removed outliers (Entamoeba in AMRO; Campylobacter, Shigella and Cryptosporidium in WPRO) were replaced by the global median etiology-proportions. | | | | | | | | | | | | |
| **Global medians estimated before a second proportion-adjustment to 100% (applied as part of the envelope-attribution step) | | | | | | | | | | | | |
| ***NA: non-applicable. Assumed to be zero. | | | | | | | | | | | | |
| ****NA: non-applicable. Calculated as the difference between the sum of all estimated etiology-proportions and 100%. | | | | | | | | | | | | |
| Table B4. Regional and global etiology-proportions of diarrhea deaths in persons >5, 2010 (median % and 95% CI).* | | | | | | | | | | | | |
| **Etiology** | | **AFRO** | | **AMRO** | | **EMRO** | | **SEARO** | | **WPRO** | | **GLOBAL**** |
| **Salmonella** | | 3.27 | | 5.25 | | 3.27 | | 3.27 | | 1.77 | | 3.27 |
|  |  | [2.85; 3.71] | | [4.17 .6.47] | | [2.85; 3.72] | | [2.84; 3.72] | | [1.30; 2.31] | | [2.85; 3.73] |
| **Campylobacter** | | 1.24 | | 2.33 | | 2.33 | | 1.25 | | 1.24 | | 1.25 |
|  |  | [1.00; 1.5] | | [1.99; 2.71] | | [2.00; 2.71] | | [1.00; 1.53] | | [1.00; 1.53] | | [1.00; 1.51] |
| **EPEC** | | 5.65 | | 5.65 | | 5.65 | | 5.65 | | 5.65 | | 5.65 |
|  |  | [4.45 ;7.06] | | [4.45; 7.01] | | [4.45; 7.08] | | [4.45; 7.07] | | [4.45; 7.05] | | [4.42; 7.04] |
| **ETEC** | | 4.09 | | 4.10 | | 4.10 | | 3.63 | | 4.54 | | 4.10 |
|  |  | [3.01; 5.42] | | [3.01; 5.40] | | [3.02; 5.39] | | [3.13; 4.17] | | [2.93; 6.65] | | [2.79; 5.24] |
| **Shigella** | | 4.13 | | 4.12 | | 4.12 | | 1.74 | | 7.97 | | 4.12 |
|  |  | [2.68; 5.97] | | [2.66; 5.99] | | [3.62; 4.68] | | [0.86; 3.12] | | [2.82; 16.55] | | [0.44; 4.65] |
| **Giardia** | | NA*** | | NA | | NA | | NA | | NA | | NA |
|  |  |  | |  | |  | |  | |  | |  |
| **Cryptosporidium** | | 1.02 | | 1.02 | | 1.01 | | 1.80 | | 0.24 | | 1.02 |
|  |  | [0.75; 1.35] | | [0.75; 1.34] | | [0.75; 1.33] | | [1.40; 2.25] | | [0.11; 0.44] | | [0.73; 1.33] |
| **Entamoeba** | | 0.22 | | 0.22 | | 0.22 | | 0.22 | | 0.22 | | 0.22 |
|  |  | [0.11; 0.40] | | [0.11; 0.40] | | [0.11; 0.40] | | [0.11; 0.40] | | [0.11; 0.40] | | [0.09; 0.39] |
| **Norovirus** | | 14.28 | | 35.98 | | 19.95 | | 19.16 | | 18.25 | | 19.15 |
|  |  | [7.90; 22.85] | | [27.17; 45.29] | | [14.46; 26.27] | | [12.56; 27.22] | | [10.59; 28.34] | | [8.66; 24.62] |
| **Unknown** | | 61.56 | | 36.84 | | 51.33 | | 62.16 | | 55.13 | | NA**** |
|  |  | [51.16; 69.50] | | [25.64; 46.76] | | [43.9; 58.27] | | [53.91; 69.20] | | [41.59; 66.40] | |  |
| **Others** | | 4.06 | | 4.12 | | 7.73 | | 0.88 | | 4.06 | | 4.08 |
|  |  | [1.03; 10.09] | | [1.06; 10.20] | | [4.69; 11.82] | | [0.32; 1.86] | | [1.11; 10.09] | | [0.00; 9.91] |
| *Missing values (Salmonella, Campylobacter, ETEC, EPEC, Shigella, Cryptosporidium, Entamoeba, and others in AFRO; ETEC, Shigella, Cryptosporidium and Entamoeba in AMRO; ETEC, EPEC, Cryptosporidium and Entamoeba in EMRO; Campylobacter, EPEC and Entamoeba in SEARO; and EPEC and others in WPRO), and removed outliers (Campylobacter in AMRO; Salmonella and Norovirus in SEARO; and Campylobacter in WPRO) were replaced by the global median etiology-proportions. | | | | | | | | | | | | |
| **Global medians estimated before a second proportion-adjustment to 100% (applied as part of the envelope-attribution step) | | | | | | | | | | | | |
| ***NA: non-applicable. Assumed to be zero. | | | | | | | | | | | | |
| ****NA: non-applicable. Calculated as the difference between the sum of all estimated etiology-proportions and 100%. | | | | | | | | | | | | |
