# Supplementary material for: Aetiology-Specific Estimates of the Global and Regional Incidence and Mortality of Diarrhoeal Diseases Commonly Transmitted through Food
Source: PLoS One. 2015 Dec 3;10(12):e0142927. doi: 10.1371/journal.pone.0142927 (PMC4668836; doi:10.1371/journal.pone.0142927)
Supplement: S1 Table — (DOCX) [file pone.0142927.s005.docx]

Table S1. Total global incidence and mortality rate (per 100,000) of 10 foodborne diarrheal pathogens (median and 95% Uncertainty Intervals).

| **Pathogen** | **INCIDENCE RATE** | | | **MORTALITY RATE** | | |
| --- | --- | --- | --- | --- | --- | --- |
|  | 61 countries in AMRO sub-region A, EURO and WPRO sub-region A) | Remaining 133 countries | Total | 61 countries in AMRO sub-region A, EURO and WPRO sub-region A) | Remaining 133 countries | Total |
| Campylobacter* | 725  (588-874) | 2,851  (1,498-5,320) | 2,415  (1,340-4,373) | 0.05  (0.02-0.1) | 0.6  (0.5-1) | 0.5  (0.4-0.8) |
| EPEC | 15  (7-26) | 1,479  (741-3,134) | 1,178  (592-2,491) | 0  (0-0)‡ | 2  (2-3) | 2  (1-2) |
| ETEC | 15  (7-26) | 4,404  (2,942-6,903) | 3,501  (2,338-5,486) | 0  (0-0)‡ | 1  (1-2) | 1  (0.8-2) |
| Norovirus** | 6,751  (5,164-8,431) | 10,764  (7,259-18,739) | 9,953  (7,135-16,320) | 0.2  (0.1-0.2) | 4  (3-5) | 3  (2-4) |
| Shigella | 60  (33-96) | 3,477  (1,774-6,648) | 2,774  (1,422-5,289) | 0.02  (0-0.06) | 1  (0.8-2) | 1  (0.7-1) |
| Salmonella*** | 323  (243-420) | 2,717  (1,108-6,906) | 2,225  (941-5,555) | 0.2  (0.1-0.3) | 1  (0.7-2) | 0.8  (0.6-1) |
| Cryptosporidium | 205  (108-360) | 1,115  (734-1,863) | 930  (626-1,521) | 0.02  (0.01-0.05) | 0.5  (0.3-0.8) | 0.4  (0.3-0.6) |
| Giardia | 384  (325-448) | 3,265  (2,279-4,711) | 2,672  (1,890-3,820) | NA^µ^ | NA^µ^ | NA^µ^ |
| Entamoeba histolytica | NA† | 1,902  (860-3,854) | 1,511  (683-3,061) | NA† | 0.1  (0.04-0.3) | 0.08  (0.03-0.2) |
| Other | NA† | 8,113  (4,857-15,176) | 6,443  (3,858-12,053) | NA† | 4  (3-5) | 3  (2-4) |
| Unknown etiology | 52,482  (39,292-66,132) | 26,669  (17,214-44,538) | 32,119  (23,941-46,234) | 1  (0.6-2) | 14  (11-16) | 11  (9-13) |
| Total | 60,981  (47,102-75,382) | 68,026  (48,996-102,730) | 66,626  (51,230-94,013) | 2  (1-2) | 29  (24-34) | 23  (20-27) |

^National studies approach applied to all countries in EURO and low mortality (WHO subregion A) countries in AMRO and WPRO.

^^ Modified CHERG approach applied to all middle/high mortality countries in AFRO, AMRO, EMRO, SEARO and WPRO.

*Diarrheal Campylobacter only (GBS-Campylobacter cases and deaths not included).

**Diarrheal norovirus only (vomiting only norovirus cases not included).

***Diarrheal non-typhoidal Salmonella only (invasive non-typhoidal Salmonella cases and deaths not included).

†Estimates not available from national studies.

‡Assumed not to cause fatalities in EURO and low mortality countries.

µAssumed not to cause fatalities.
